# Molecular Engineering of Ionic Metal‐Organic Frameworks via Ligand Conjugation Modulation for Tailored Phosphorescence and Multilevel Encryption

**DOI:** 10.1002/advs.202509013

**Published:** 2025-07-12

**Authors:** Qi Wu, Xinyu Gao, Wenyan Zhang, Yaoyu Wang

**Affiliations:** ^1^ Key Laboratory of Synthetic and Natural Functional Molecule of the Ministry of Education College of Chemistry & Materials Science Northwest University Xi'an 710127 P. R. China

**Keywords:** information encryption, ionic metal‐organic framework, ligand conjugation, room‐temperature phosphorescence, structural regulation

## Abstract

The rational design of metal‐organic frameworks (MOFs) with tunable phosphorescence remains challenging due to limited understanding of ligand conjugation effects on excited‐state dynamics. Herein, two isomorphic ionic MOFs (IMOF‐DMF and IMOF‐DEF) are constructed via in situ decarbonylation of DMF/DEF, leveraging guest‐ion size to regulate ligand conjugation through modulation of pyridine ring dihedral angles. IMOF‐DEF exhibits superior room‐temperature phosphorescence (RTP) and temperature‐dependent behavior. Mechanistic studies reveal that reduced conjugation enhances intersystem crossing (ISC) by strengthening spin‐orbit coupling while minimizing the singlet‐triplet energy gap (ΔE_st_). Furthermore, a dual‐parameter anti‐counterfeiting platform and flexible PVA hydrogel films enable advanced information encryption through temperature/time‐resolved signals and triple‐state lifetime encoding. This work establishes ligand conjugation engineering as a key strategy for tailoring MOF photophysics, linking molecular design to optoelectronic applications and security technologies.

## Graphical abstract

This work exhibits a molecular engineering approach for efficient equilibrium phosphorescence through guest ion‐mediated ligand conjugation to modulate ionic Metal‐Organic Frameworks excited state dynamics. The designed IMOF‐DMF realizes temperature‐responsive room‐temperature phosphorescence and multi‐state lifetime encoding for anti‐counterfeiting and is supported by experimental and theoretically calculated mechanisms.

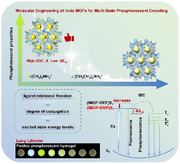

## 1 Introduction

Phosphorescent materials have shown irreplaceable advantages in bio‐imaging,^[^ ^1^, ^2^, ^3^, ^4^, ^5^, ^6^, ^7^, ^8^ ^]^ anti‐counterfeiting techniques,^[^ ^9^, ^10^, ^11^, ^12^, ^13^ ^]^ optical sensing,^[^ ^14^, ^15^, ^16^, ^17^, ^18^, ^19^ ^]^ and light‐emitting diodes (LEDs)^[^ ^20^, ^21^, ^22^ ^]^ by its unique long‐lived luminescence characteristics and customisable optical properties. However, the room‐ and long‐term phosphorescence (RTP) at room temperature faces many challenges: on the one hand, non‐radiative transition, such as intermolecular collisions and energy transfer, occur frequently at room temperature, which affects the efficiency of phosphorescence^[^ ^23^, ^24^, ^25^ ^]^; on the other hand, triplet excitons are extremely sensitive to the environment (e.g., oxygen, temperature, humidity) and are vulnerable to energy dissipation through non‐radiative pathways, which leads to the difficulties of ‘long lifetime – low efficiency’ in most systems.^[^ ^26^, ^27^, ^28^ ^]^ In order to solve the above problem, researchers usually enhance the spin‐orbit coupling by introducing heavy atoms to improve the intersystem crossing efficiency.^[^ ^29^, ^30^, ^31^ ^]^ Secondly, synthesising crystalline materials to inhibit the non‐radiative transitions of triplet excitons is also an effective strategy to improve the phosphorescence quantum yield.^[^ ^32^, ^33^, ^34^, ^35^ ^]^ In addition, long‐lived room‐temperature phosphorescence can be realised by embedding phosphorescent groups into solid matrices to cut off outside environments and other factors and immobilise their structures to limit non‐radiative decay.^[^ ^36^, ^37^, ^38^, ^39^ ^]^ However, these methods often limit their applications due to poor material processing or ignoring structure structure‐activity relationship.

Metal‐organic Frameworks (MOFs) have been attracting significant attention in the field of phosphorescent materials due to their high crystallinity, rigid frameworks, and periodic pore structures, which provide new ways of balancing the phosphorescence lifetime with environmental stability.^[^ ^40^, ^41^ ^]^ Stable connections between metal nodes and ligands in the framework confer rigidity to the whole structure, greatly reducing the non‐radiative leaps.^[^ ^42^, ^43^ ^]^ Traditional MOFs materials have demonstrated tunable phosphorescence.^[^ ^44^, ^45^, ^46^ ^]^ However, the precise regulation of phosphorescence through dynamic control of ligand conjugation mediated by guest‐ion interactions, particularly in ionic MOF systems, remains underexplored, limiting the rational design of materials with tailored excited‐state dynamics.

In this work, Zn(II) and 2‐(3,5‐dicarboxyphenyl) nicotinic acid (H_3_L) ligand were used as the node and linker, respectively, to successfully construct two examples of isostructural IMOFs (**IMOF‐DMF**, **IMOF‐DEF**) via solvothermal in situ decarbonylation reaction. The microscopic mechanism governing the modulation of spin‐orbit coupling (SOC) strength and singlet‐triplet energy gap (ΔE_st_) via structural torsion was revealed through a comparative analysis of conjugation changes caused by differences in pyridine ring rotational freedom between **IMOF‐DMF** and **IMOF‐DEF**. Further, to focus on the environmental sensitivity of phosphorescent materials, a polyvinyl alcohol (PVA) hydrogel encapsulation strategy was proposed, and phosphorescent films were successfully constructed. Based on the lifetime encoding property of triple‐state excitons, an anti‐counterfeit encryption system was developed, which provides an experimental basis for the application of smart luminescent materials in the fields of flexible optoelectronics and information security.

## 2 Results and Discussion

### 2.1 Synthesis and Characterization of Crystal

The crystal structure of **IMOF‐DMF** is in the orthorhombic crystal system with the space group *P*2_1_2_1_2. the unit of asymmetry consists of two H_3_L ligands, one Zn(II), and two free (CH_3_)_2_NH_2_^+^. As shown in Figure S3a (Supporting Information), a Zn(II) ion is coordinated by four differently oriented L_3_^−^ ligands to form a 3D network structure. The framework can be simplified into a *pcl* topology with the topological symbol [4^2^·6^3^·8] (Figure S3d, Supporting Information). The structure exists ionic pores with a pore size of 7.4 × 5.6 Å^2^ along the *a* axis (Figure S3b,c, Supporting Information), and the porosity of **IMOF‐DMF** was calculated to be 13.1% using the PLATON program. **IMOF‐DEF** is coordinated in the same way as **IMOF‐DMF**. They are isostructural crystals, so the simplified topological model of **IMOF‐DEF** is also the *pcl* topology of [4^2^·6^3^·8], and the porosity of this IMOF is only 2.7% calculated by the PLATON program. Notably, the presence of (CH_3_CH_2_)_2_NH_2_^+^ in the ionic pore channel of **IMOF‐DEF** with a larger size performs an important role in balancing the framework charge. Compared with (CH_3_)_2_NH_2_^+^ in **IMOF‐DMF**, the dihedral angle between the benzene and pyridine rings in the ligand is smaller because (CH_3_CH_2_)_2_NH_2_^+^ occupies more space in the ionic pores, which makes the rotational space of the pyridine ring further restricted. The dihedral angle between the benzene ring and the pyridine ring in **IMOF‐DEF** is 60.67° after single crystal structure analysis, while this dihedral angle is 58.67° in **IMOF‐DEF** (Figure (**1a,b**))(II).

**Figure 1:**
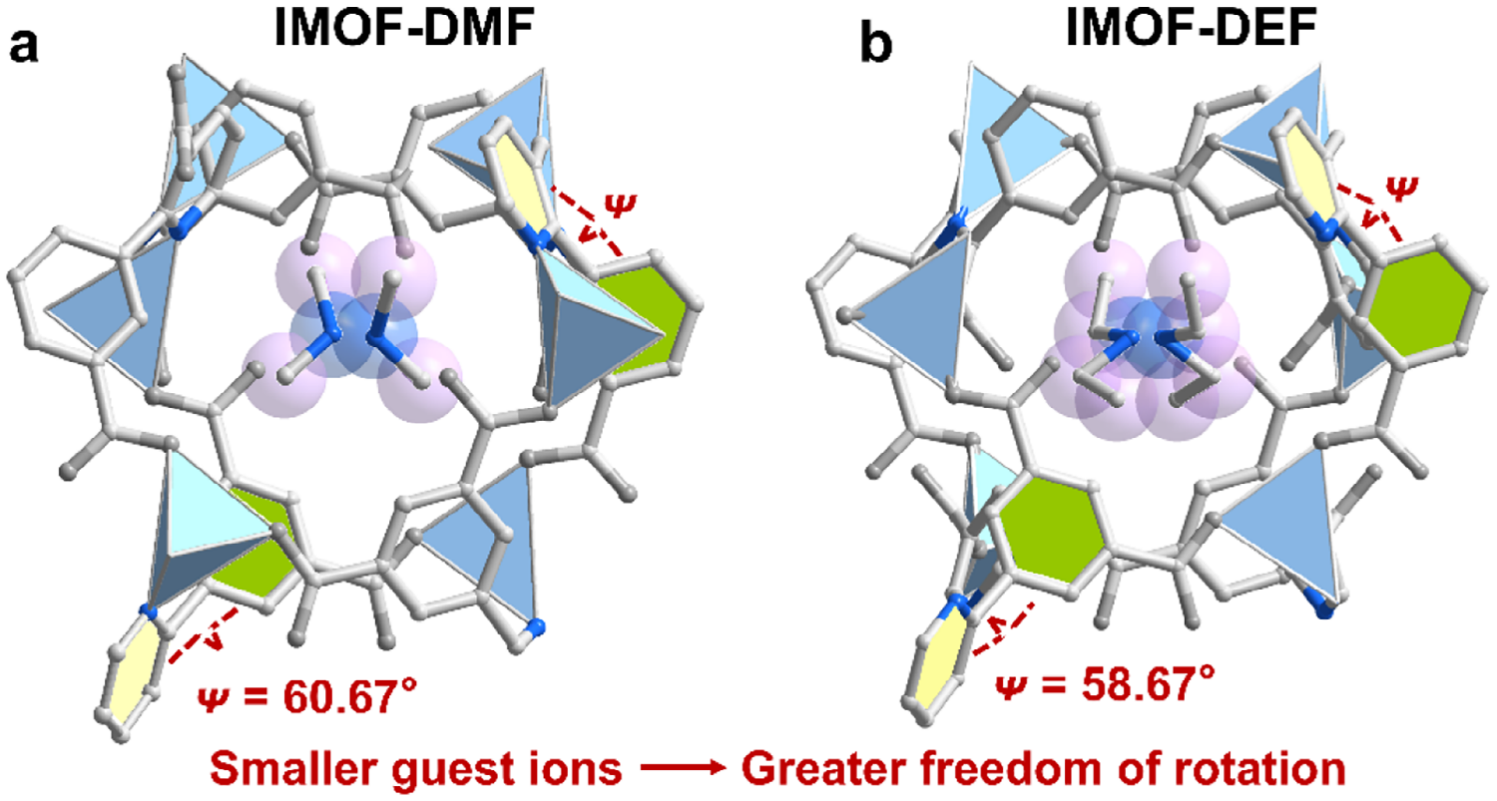
a) **IMOF‐DMF** and b) **IMOF‐DEF** structure of the pore channel.

**Figure 2:**
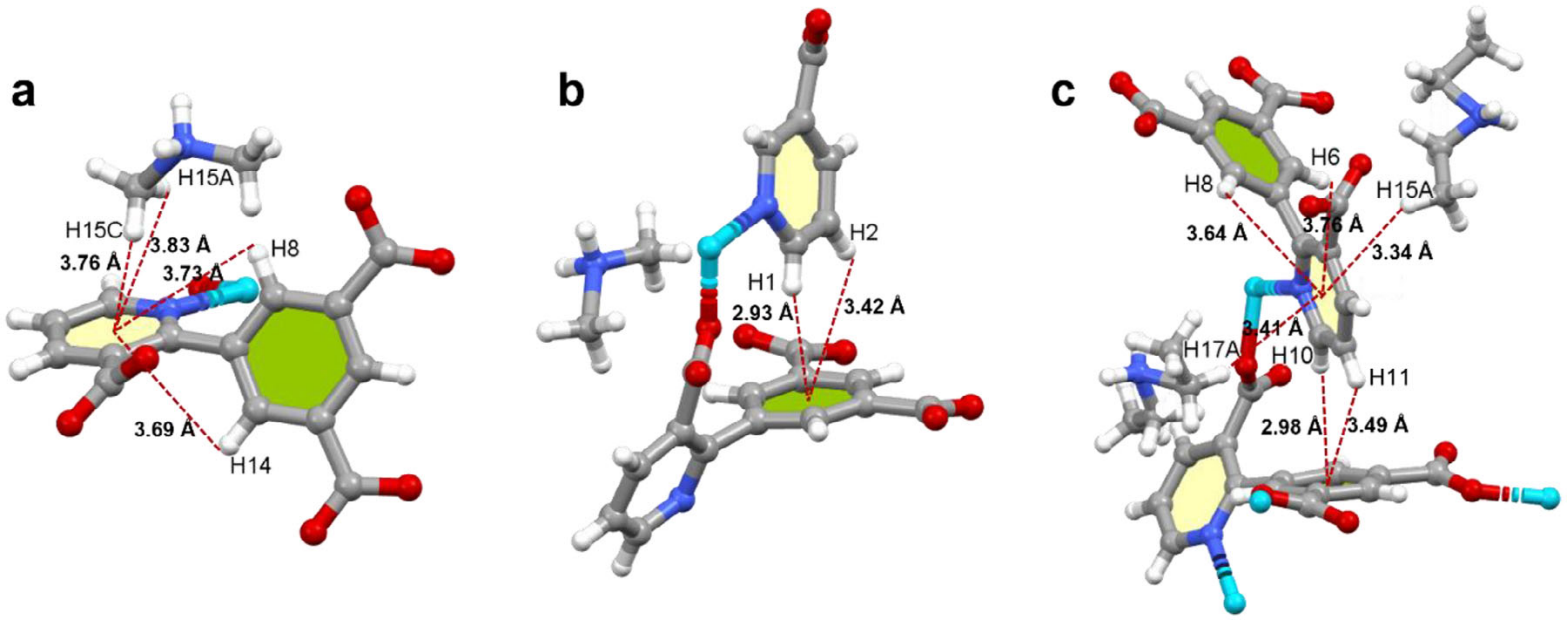
**IMOF‐DMF** in a) cation‐*π* interactions of the pyridine ring with the (CH_3_)_2_NH_2_^+^ cation and conjugation effects of the pyridine ring‐benzene ring H within the same ligand; b) C‐H···*π* interactions of the pyridine ring H – benzene ring between neighboring ligands; and c) multiple interactions in the **IMOF‐DEF**.

The difference in dihedral angle affects the intramolecular/intermolecular interactions in the two examples of IMOFs. X‐ray single crystal diffraction analysis reveals a variety of the same types of interactions in the **IMOF‐DMF** and **IMOF‐DEF** systems: i) cation‐π interactions between the pyridine ring and the guest cation; ii) C‐H···*π* interactions of the pyridine ring benzene ring between neighbouring ligands; and iii) intra‐ligand pyridine ring–benzene ring H conjugation effect Figure (**2a,b**). However, the increased dihedral angle within the **IMOF‐DMF** ligand promotes the superior face‐to‐edge orientation of the pyridine ring to the benzene ring between the neighbouring ligands, with shorter bond lengths for the C‐H···*π* interactions (2.93, 3.42 Å) than those of the **IMOF‐DEF** (2.98, 3.49 Å), suggesting that the former has a higher strength of the noncovalent interactions (Figure 2c). On the one hand, the stacking of face‐to‐edge orientation leads to a more stable structure^[^ ^47^ ^]^; on the other hand, tighter intermolecular interactions may promote electron transfer between molecular orbitals, which usually enhances the SOC process and thus may facilitate the generation of phosphorescence.^[^ ^48^, ^49^ ^]^

It can be seen from the scanning electron microscope (SEM) spectrum, IMF‐DMF is a rectangular block of about 50 µm, while IMF‐DEF is a square block of ≈30 µm (Figure S4, Supporting Information). The phase purity of the samples was analyzed by X‐ray powder diffraction (PXRD). As shown in Figure S5a (Supporting Information), the PXRD patterns of the synthesised **IMOF‐DMF** and **IMOF‐DEF** are in good agreement with the data from the single‐crystal structure simulation, thus proving that **IMOF‐DMF** and **IMOF‐DEF** are pure phases. It is worth noting that due to the larger guest ions in the **IMOF‐DEF** channel occupying a larger lattice position, the crystal cell is slightly deformed compared to **IMOF‐DMF**, which further affects the position of the PXRD diffraction peak, resulting in a 0.56° shift to a lower Angle (Figure S5b, Supporting Information). In addition, when two IMOFs were immersed in aqueous solutions of different pH and in different solvents, the two IMOFs still maintained good crystallinity, which proved that **IMOF‐DMF** and **IMOF‐DEF** had good chemical stability (Figures S6 and S7, Supporting Information).

Thermogravimetric (TGA) was used to analyse the stability of two IMOFs. As shown in Figure S8a (Supporting Information), under N_2_ atmosphere, **IMOF‐DMF** showed a weight loss of about 2.7% in the 30–150°C interval, corresponding to the release of guest water molecules in the MOF framework. The subsequent 3.5% weight loss ≈200–250°C corresponds to the loss of (CH_3_)_2_NH_2_^+^ in the **IMOF‐DMF** framework. **IMOF‐DMF** is thermally stable up to 370°C, and the framework subsequently begins to collapse as the temperature increases. As shown in Figure S8b (Supporting Information), there does not seem to be any release of guest solvents from **IMOF‐DEF**, which may be due to the fact that the pores of **IMOF‐DEF** are small and occupied by (CH_3_CH_2_)_2_NH_2_^+^, whereas the DEF solvent molecules are larger and more difficult to enter the pores.

### 2.2 Photophysical Characterization of IMOFs

**Figure 3:**
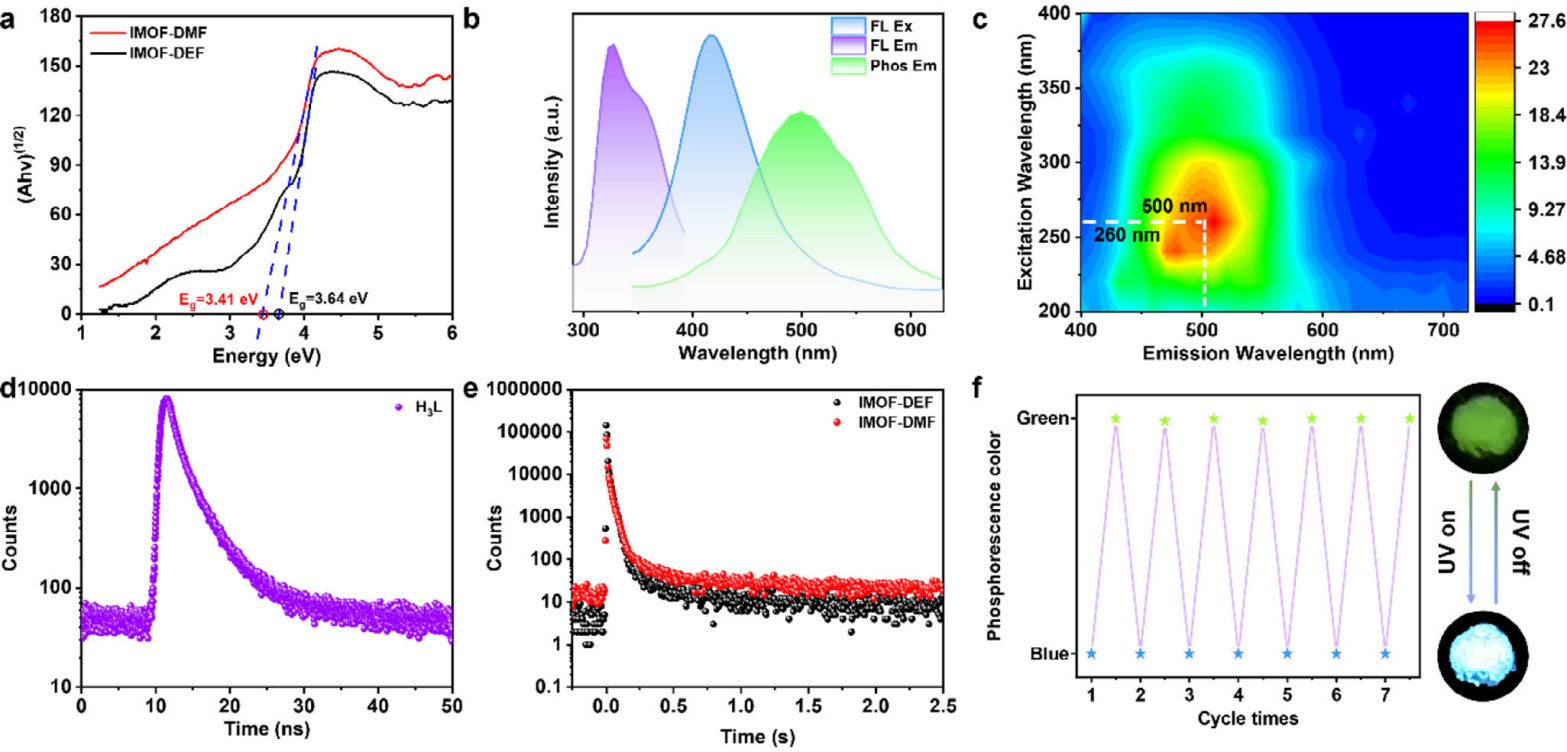
Photophysical characterization of IMOFs a) Optical bandgap; b) PL spectra of IMOF‐DMF; c) 3D phosphorescence mapping; d) Phosphorescence decay profiles of H_3_L ligand; e) Phosphorescence decay profiles of IMOFs (RT); f) Luminescence cycling test of **IMOF‐DMF**.

The relationship between the photophysical properties of two isomorphic IMOFs and their structural differences is investigated in detail. By solid‐state ultraviolet‐visible diffuse reflectance spectroscopy (UV–vis DRS) analysis (Figure S9, Supporting Information), it was observed that **IMOF‐DMF** and **IMOF‐DEF** exhibited significant UV absorption peaks attributed to π‐π* transition near 280 and 290 nm, respectively.^[^ ^50^, ^51^ ^]^ The optical bandgaps of the two materials were further calculated by the Tauc plot method (**Figure** **3a**), which showed that the bandgap value of **IMOF‐DMF** was 3.41 eV, whereas the bandgap of **IMOF‐DEF** was slightly wider (3.64 eV). This difference indicated that the energy level difference between the top of the Valence Band Maximum (VBM) and the Conduction Band Minimum (CBM) in **IMOF‐DMF** is smaller, and its electrons are more prone to transit from the valence band to the conduction band, which can significantly enhance the excitation probability.^[^ ^52^ ^]^

The H_3_L ligand and its assembled Zn(II)‐based ionic coordination polymers (**IMOF‐DMF** and **IMOF‐DEF**) were investigated by photoluminescence spectroscopy (PL) at room temperature to study their luminescence behaviour and mechanism. The results showed that the H_3_L ligand exhibited typical fluorescence emission characteristics at an excitation wavelength of 248 nm, with its maximum emission wavelength located ≈400 nm. In addition, a weak phosphorescence emission was observed at 525 nm for H_3_L, but due to its low intensity, it was almost impossible to be directly observed by the naked eye (Figure S10, Supporting Information). This weak phosphorescence phenomenon may be related to the lack of rigid structure of the H_3_L molecule in its free state.

The luminescent properties of **IMOF‐DMF** and **IMOF‐DEF** changed significantly when the H_3_L ligands were coordinated with Zn(II) ions to form **IMOF‐DMF** and **IMOF‐DEF**. As shown in Figure 3b, **IMOF‐DMF** exhibited strong blue fluorescence emission at an excitation wavelength of 328 nm, and its maximum emission wavelength was located at 416 nm, corresponding to the CIE chromaticity coordinate of (0.162, 0.081). Emission spectra of **IMOF‐DMF** under different solvents demonstrated that it possessed solvent‐dependent photoluminescence behavior. As shown in Figure S11 (Supporting Information), when MeOH is used as the solvent, the increase in solvent polarity leads to a decrease in the excited state energy by enhancing the stability of the charge transfer (CT) excited state, and the maximum emission peak of **IMOF‐DMF** is red‐shifted to ≈420 nm compared to that of the solid.^[^ ^53^, ^54^ ^]^ After turning off the excitation light source, **IMOF‐DMF** also exhibits a green afterglow phenomenon visible to the eye. The maximum wavelength of its phosphorescence emission was further confirmed by phosphorescence spectroscopy tests to be located at 500 nm, corresponding to the CIE chromaticity coordinate of (0.205, 0.382) (Figure 3c). Similarly, **IMOF‐DEF** exhibited blue fluorescence emission at an excitation wavelength of 332 nm, with a maximum emission wavelength located at 427 nm and CIE chromaticity coordinate of (0.159, 0.081), and a green afterglow was observed after switching off the light source. Phosphorescence spectroscopy showed that the maximum wavelength of phosphorescence emission of **IMOF‐DEF** was located at 514 nm with CIE chromaticity coordinates of (0.215, 0.473) (Figure S12a,b, Supporting Information). The optimal fluorescence emission peaks of **IMOF‐DMF** and **IMOF‐DEF** exhibit a redshift of 15–30 nm compared to the H_3_L ligand. This confirms that the fluorescence originates from the ligand‐centered emission of H_3_L, and the redshift is induced by energy level splitting resulting from ligand‐metal coordination. The fluorescence lifetime of H_3_L was calculated to be 2.44 ns based on the PL decay curve at room temperature (Figure 3d), while the phosphorescence lifetimes of **IMOF‐DMF** and **IMOF‐DEF** were 206 and 173 ms, respectively (Figure 3e). The RTP quantum yields measured using the absolute method with an integrating sphere, are 12.27% for **IMOF‐DMF** and 1.76% for **IMOF‐DEF**. In addition, as shown in Figure 3f, after seven repeated irradiations with 365 nm UV light, the samples consistently retained their original photophysical properties and exhibited excellent recyclability. Furthermore, the comparisons of **IMOF‐DMF** and **IMOF‐DEF** with other materials (Table S4, Supporting Information). Overall, **IMOF‐DMF** has an ultra‐long afterglow of ≈3 s, while maintaining a long lifetime and a high quantum yield. These results are significantly better than those of most reported MOF‐based phosphorescent materials.

Compared with the H_3_L ligand, **IMOF‐DMF** and **IMOF‐DEF** exhibit significantly enhanced phosphorescence properties, which may be attributed to several factors: firstly, the coordination of Zn(II) metal ions effectively enhances the rigidity of the ionic framework, which inhibits the non‐radiative leap process caused by molecular vibrations and rotations, and reduces the energy loss and promotes the stabilisation of triplet state excitons.^[^ ^55^ ^]^ Secondly, the metal ion introduction may further modify the energy distribution of the excited state and enhance the ISC efficiency through the ligand‐to‐metal charge transfer (LMCT) or metal‐to‐ligand charge transfer (MLCT) mechanism.^[^ ^56^ ^]^ In addition, the guest ions in IMOFs may also reduce the bursting effect of oxygen on triplet‐state excitons through the pore domain‐limiting effect, further extending the lifetime of phosphorescence.^[^ ^57^ ^]^

**Figure 4:**
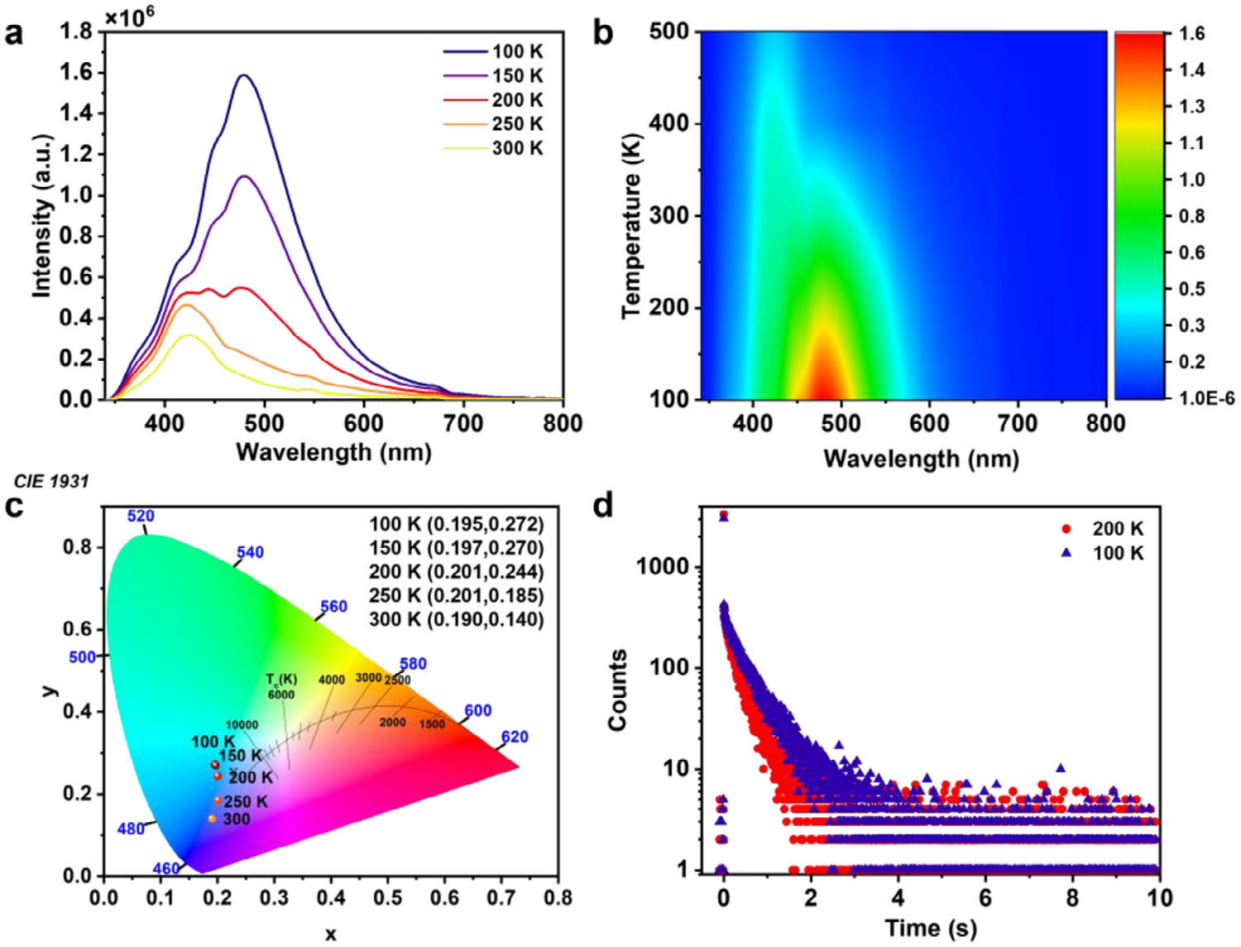
PL spectra of **IMOF‐DMF**. a) Typical curves; b) 2D contours, and c) corresponding CIE coordinates for the temperature‐dependent emission of **IMOF‐DMF** at the excitation wavelength of 328 nm; d) Phosphorescence decay curves of **IMOF‐DMF** at 100 and 200 K.

In order to further elucidate the photophysical properties of the two examples of IMOFs, the PL spectra and PL decay curves of **IMOF‐DMF** and **IMOF‐DEF** with temperature were systematically investigated in order to reveal their excited state kinetic behaviour and the mechanism of the temperature effect on the luminescence performance. As shown in  Figures **4a–c** and S13a–c (Supporting Information), the solid‐state **IMOF‐DMF** exhibits a multi‐emission band feature at 100 K. The solid‐state **IMOF‐DMF**s are also characterised by the high temperature of the excited state and the high temperature of the excited state. With the gradual increase of temperature from 100 K to room temperature 300 K, the intensity of the long wavelength phosphorescence peaks of **IMOF‐DMF** was significantly weakened, and also its phosphorescence lifetime showed an obvious decreasing trend. As shown in Figure 4d, the phosphorescence decay curves of **IMOF‐DMF** exhibit a significant temperature dependence at the excitation wavelength of 326 nm. As the temperature decreases from 200 to 100 K, its phosphorescence lifetime is significantly extended from 269 to 566 ms. This significant increase in lifetime is mainly attributed to the inhibition of the vibrational relaxation (VR) process at low temperatures, which reduces the competition for non‐radiative leaps, improves the ISC efficiency, and stabilises the triple‐state exciton lifetime.^[^ ^58^ ^]^ In addition, the significant prolongation of the phosphorescence lifetime at low temperatures may indicate the participation of higher‐order triplet states, which are more prone to accumulate excitons at low temperatures due to their larger energy gaps.^[^ ^59^ ^]^ Furthermore, with the increase of temperature, **IMOF‐DMF** gradually changed from phosphorescence dominated by green (0.195,0.272) to fluorescence dominated by blue (0.190,0.140), which further confirms the temperature‐adjusted effect on the energy distribution of excited states.

### 2.3 Theoretical Calculations

**Figure 5:**
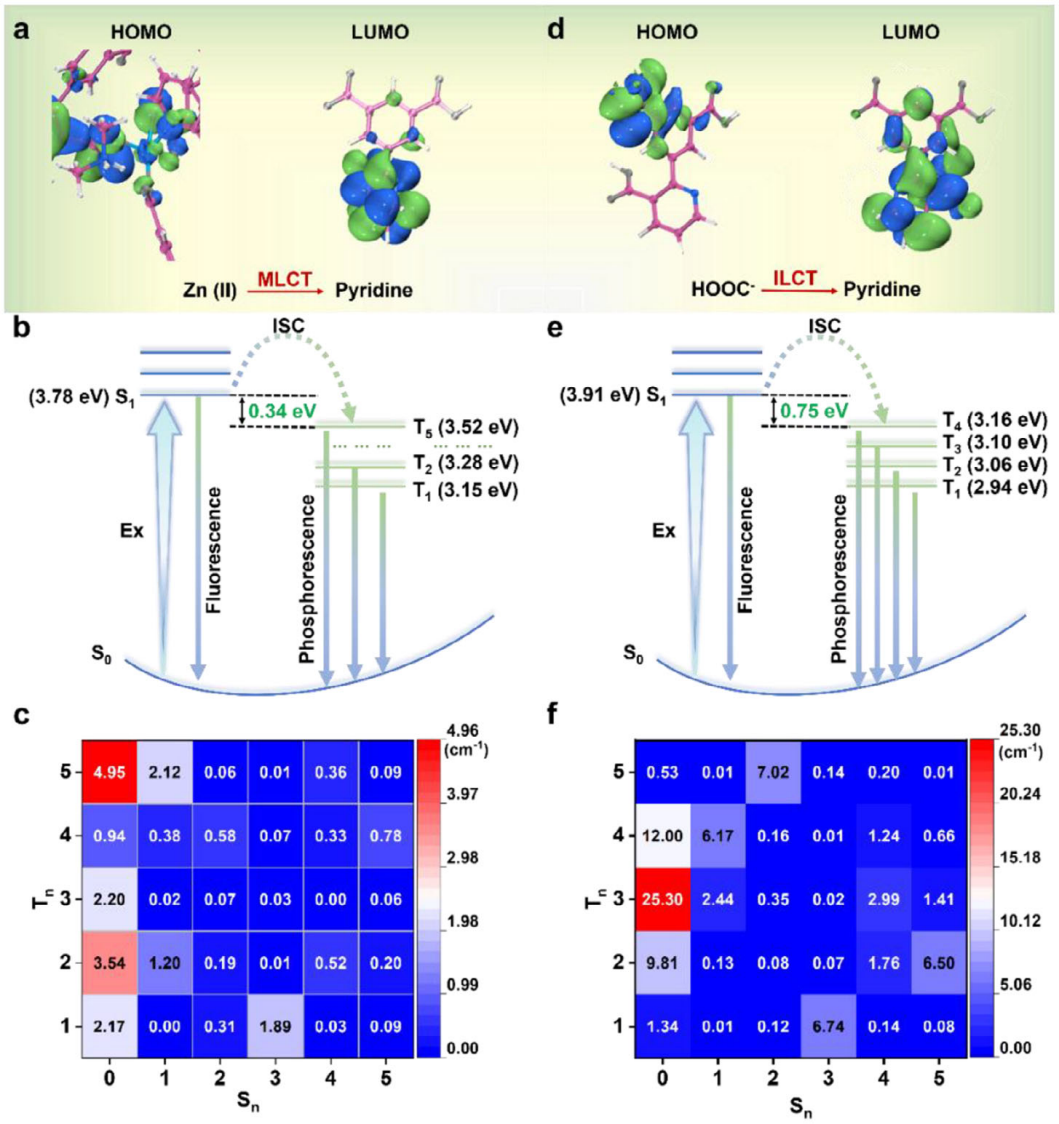
TD‐DFT calculations and energy level diagram. Frontier molecular orbital distributions for selected segments from a) **IMOF‐DMF** and d) **IMOF‐DEF**; energy levels of the singlet and triplet excited states of b) **IMOF‐DMF** and e) **IMOF‐DEF**; SOC matrix element heat maps for c) **IMOF‐DMF** and f) **IMOF‐DEF**.

In order to investigate the differences in the luminescence mechanisms of **IMOF‐DMF** and **IMOF‐DEF**, an excited‐state dynamics investigation has been carried out systematically. A coordination centre interception strategy was utilised: the guest ion and the four organic ligands coordinated to the Zn(II) centre were retained, and a simplified computational model was constructed through the hydrogen atom saturated boundary. The frontline molecular orbitals (HOMO, highest occupied molecular orbital; LUMO, lowest unoccupied molecular orbital) of **IMOF‐DMF** and **IMOF‐DEF** are shown in  Figure **5a,d**. The results show that the HOMO of **IMOF‐DMF** is mainly occupied by Zn(II) and HOOC^−^ coordinated to it, whereas the LUMO consists mainly of the pyridine ring contribution of the H_3_L ligand. The electron distribution characteristics indicate the presence of MLCT‐dominated excited states in the system and exhibit significant charge separation properties (electron transfer from the Zn(II) centre to the H_3_L ligand).^[^ ^60^ ^]^ This charge separation character may inhibit electron‐hole complexation, thereby prolonging the excited state lifetime and reducing non‐radiative leaps.^[^ ^61^, ^62^, ^63^ ^]^ In contrast, the HOMO of **IMOF‐DEF** is mainly distributed on the carboxylic acid and its connected benzene ring, and the LUMO consists of the contribution from the pyridine ring. This electron distribution feature indicates the presence of an excited state dominated by Intraligand Charge

Transfer (ILCT) in the system. The charge transfer range of ILCT tends to be limited, and electron‐hole pairs are likely to complex via non‐radiative pathways, leading to shortened excited state lifetimes. The vertical excitation energy of S_0_→S_1_ of **IMOF‐DMF** is 3.78 eV (corresponding to the wavelength of 305 nm), which is in good agreement with the peak of its experimental solid‐state absorption spectrum at 295 nm, verifying the reliability of the calculated model. The calculated phosphorescence emission energy level of the triple state shows a significant energy level splitting phenomenon (Figure 5b), in which the calculated value of the phosphorescence emission energy level of T_5_‐S_0_ is 3.52 eV. TD‐DFT theoretical calculations are performed by ORCA software to obtain the spin‐orbit coupling for the closed‐shell layer system. As shown in Figure 5c, ζ(S_1_,T_5_) = 2.12 cm^−1^ is significantly higher than other ζ(S_1_,T_n_) values, while the ΔE_st_ is 0.34 eV, which satisfies the thermodynamic requirement of El‐Sayed rule for efficient ISC. In summary, the synergistic effect of the stronger SOC coupling between the S_1_ and T_5_ states and the narrower energy gap provides a dual promotion mechanism for the ultrafast ISC process.

In contrast, theoretical calculations of the **IMOF‐DEF** system show that its S_0_→S_1_ vertical excitation energy is 3.91 eV (corresponding to a wavelength of 316 nm), which is blue‐shifted by 0.13 eV compared with that of **IMOF‐DMF**. This may be due to the fact that the larger guest ions in **IMOF‐DEF** limit the rotational degrees of freedom of the pyridinium ring, leading to a decrease in the angle of rotation, which strengthens the degree of conjugation of the system and enhances the excited state energy level. As shown in Figure 5e, the calculated values of the phosphorescence emission energy levels further reveal the triple‐state features: T_4_→S_0_(3.16 eV), T_3_→S_0_(3.10 eV), T_2_→S_0_(3.06 eV), and T_1_→S_0_(2.94 eV), which show a downward shifting trend of the energy levels. Notably, the SOC matrix analysis shows that ζ(S_1_,T_4_) = 6.17 cm⁻^1^ is larger than other ζ(S_1_,T_n_) values as well as the **IMOF‐DMF** system (Figure 5f). Therefore, although ΔE_st_ = 0.75 eV is higher than that of the **IMOF‐DMF** regime, the higher SOC constant of **IMOF‐DEF** implies that the mixing of spin angular momentum with orbital angular momentum is more efficient, which helps to promote the ISC process.^[^ ^64^, ^65^ ^]^ As a result, **IMOF‐DEF** exhibits weaker phosphorescent properties at room temperature.

### 2.4 RTP for Optical Anti‐Counterfeiting and Encryption

**Figure 6:**
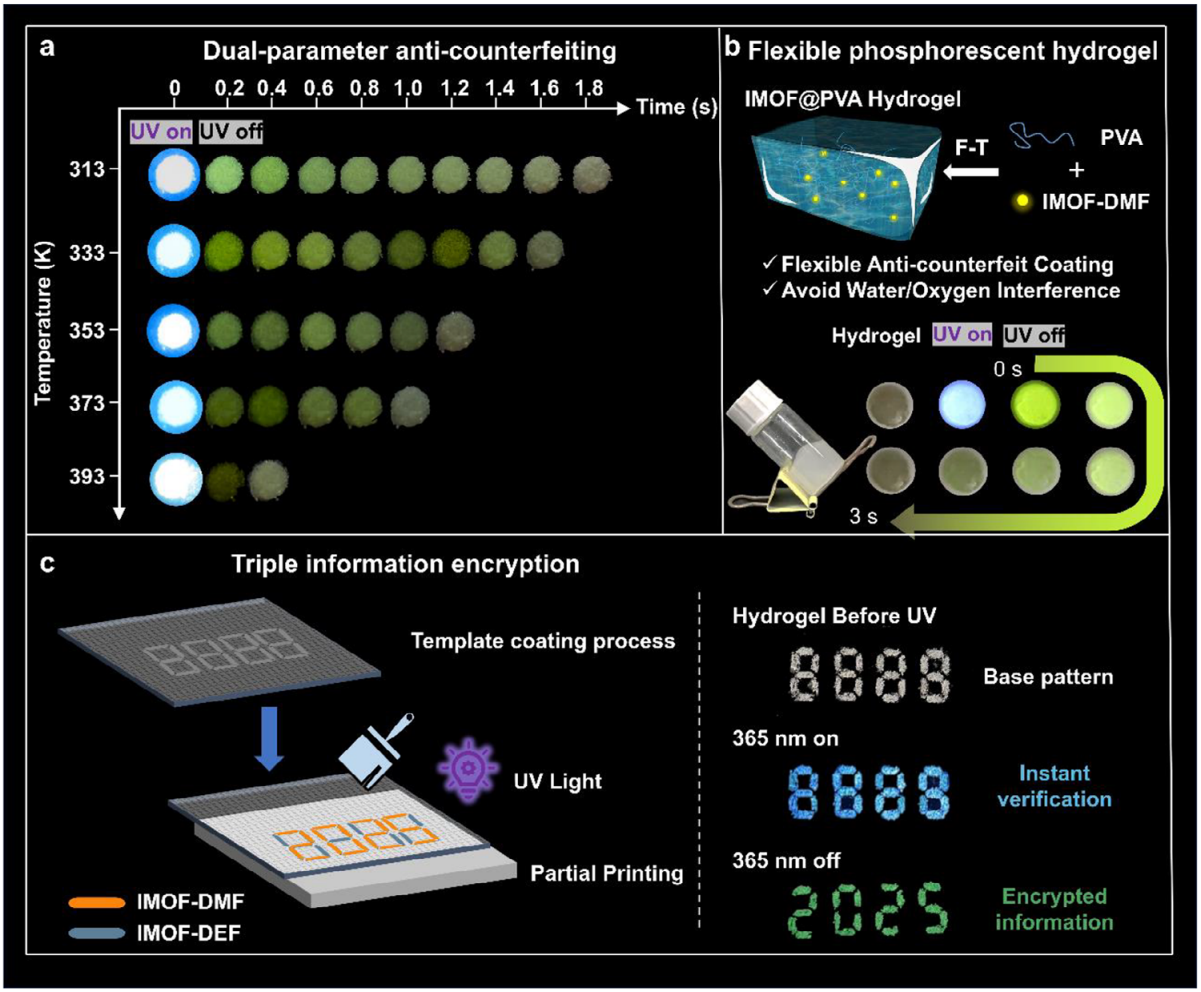
a) The temperature‐related dual‐parameter anti‐counterfeiting application of IMOF‐DMF; b) IMOF@PVA hydrogel and its RTP photographs; c) Triple message encryption by step‐by‐step template coating process.

Given the unique response of **IMOF‐DMF** to temperature and the differential phosphorescence lifetime of the two examples of IMOFs, the potential applications of **IMOF‐DMF** in areas such as optical anti‐counterfeiting and encryption were further investigated. Similar to the above phenomenon, from the perspective of time, after removing the UV light source, the **IMOF‐DMF**s showed a time‐dependent RTP from green to white at 313 K, while the intensity of the RTP gradually weakened and the color tended to become lighter as the temperature increased. Compared with the traditional single‐parameter dependent anticounterfeiting materials, this time‐ and/or temperature‐dependent dual‐parameter anti‐counterfeiting material has higher imitation difficulty, which makes it have a wide range of application prospects (**Figure** **6a**).

Polyvinyl alcohol (PVA) hydrogels have good film‐forming properties and easy processing, which can be prepared into different forms of films and coatings by simple processes, achieving effective coating of complex structural materials and facilitating the application to the surfaces of different anti‐counterfeiting materials.^[^ ^66^ ^]^

Compared with pure MOFs materials, IMOF@PVA composites can significantly improve the surface coverage uniformity and form a continuous phosphorescent anti‐counterfeiting coating with good adhesion. Therefore, IMOF@PVA hydrogels were prepared by the classical freeze‐thaw (F‐T) method, which not only can effectively encapsulate the phosphorescent IMOFs to prevent the interference of water/oxygen environment, but also endow the materials with excellent flexible machining properties. As shown in Figure 6b and Figure S14 (Supporting Information), the IMOF@PVA hydrogel appears as a colorless transparent gel under no UV light source and emits blue fluorescence under 365 nm UV light source. When the UV light is turned off, the hydrogel fluoresces green and shows an afterglow of ≈3 s. This dynamic optical property provides a technical basis for multiple anti‐counterfeiting verifications.

In the application of information encryption (Figure 6c), a triple information encryption system has been successfully constructed by precisely regulating the phosphorescent properties of **IMOF‐DMF** and **IMOF‐DEF** and combining with the template coating process: Display the white “8888” base pattern in the absence of light; Under UV light source, it presents a blue “8888” instant verification message. After turning off the ultraviolet light, the “2025” encrypted information was interpreted through the difference in phosphorescence lifetime. This time and/or graphically resolved encryption strategy significantly increases the decoding complexity and imitation difficulty of the anti‐counterfeiting system.

## 3 Conclusion

In summary, the in situ decarbonylation of DMF/DEF was achieved to generate (CH_3_)_2_NH_2_^+^/(CH_3_CH_2_)_2_NH_2_^+^ guest ions, and two isomorphic IMOFs (**IMOF‐DMF** and **IMOF‐DEF**) were successfully constructed based on this template. Although both have the same topo network, the guest ion volume difference ((CH_3_CH_2_)_2_NH_2_^+^>(CH_3_)_2_NH_2_^+^) significantly modifies the rotational freedom of the pyridine ring in the ligand: the smaller (CH_3_)_2_NH_2_^+^ template in **IMOF‐DMF** induces the ligand to form a larger dihedral angle (ψ = 59.88°), resulting in a lower conjugation degree compared with **IMOF‐DEF** (ψ = 56.99°). The photophysical study confirms that the **IMOF‐DEF** with a lower conjugation degree exhibits superior room temperature phosphorescence performance, with a phosphorescence lifetime of up to 206 ms at 300 K and a significant temperature‐dependent property (T_300 K_/T_100 K_ = 0.36). Through theoretical calculation and analysis, the characteristics of the excited state dominated by MLCT in the **IMOF‐DMF** system were revealed. Furthermore, the reduction of the conjugation degree induced a synergistic effect between the strong SOC constant ξ = 2.12 cm^−1^ and the small ΔE_st_(S_1_‐T_5_) = 0.34 eV, providing a dual promoting mechanism for faster ISC. In view of the unique response of **IMOF‐DMF** to temperature, a time and/or temperature‐dependent two‐parameter anti‐counterfeiting platform is designed. In view of the unique response of **IMOF‐DMF** to temperature, a time and/or temperature‐dependent two‐parameter anti‐counterfeiting platform is designed. To address the environmental sensitivity of phosphorescent materials, the PVA hydrogel encapsulation strategy was further designed to construct flexible phosphorescent films with continuous interfaces, and the higher‐order information encryption was realized based on the principle of triple‐state lifetime encoding. This work reveals the regulation law of the ligand conjugation degree on the excited state dynamics of MOFs through precise design strategies, and successfully expands the application scenarios of IMOFs in the intersection of flexible optoelectronic devices and quantum information encryption.

## 4 Experimental Section

### Apparatus and Materials

Fourier Transform infrared spectroscopy (FT‐IR) was collected using a Bruker EQUINOX‐55 spectrophotometer in the 4000–400 cm^−1^ range. A Bruker D8 ADVANCE X‐ray powder diffractometer (Cu Kα, 1.5418 Å) was used to record the Powder X‐ray diffraction (PXRD) pattern. Under a N_2_ atmosphere, thermogravimetric analyses (TGA) were carried out on the NETZSCH STA 449C microanalyzer thermal analyzer. All the relevant Photoluminescence Spectroscopy (PL) tests, including fluorescence, phosphorescence, and time‐resolved PL lifetime, were conducted on an Edinburgh FLS920 and FLS980 fluorescence spectrometer.

2‐(3,5‐dicarboxyphenyl) nicotinic acid (H_3_L, 97.0%), uric acid, polyvinyl alcohol (type 1799) were purchased from Macklin Biochemical Co. Ltd. All chemicals were used without further purification. All experiments were prepared using ultrapure water.

### Synthesis of **IMOF‐DMF**

14 mg of Zn(NO_3_)_2_·6H_2_O with 5.7 mg of H_3_L ligand was added to 2 mL of DMF solvent and ultrasounded for 15 min to obtain a clear solution. Subsequently, 50 µL of nitric acid was added to this solution, and the mixed solution was transferred to a 25 mL teflon reactor and reacted at 105 °C for 3 days. At the end of the reaction, the product was washed by using DI water, and finally, transparent bulk crystals of **IMOF‐DMF** were obtained by centrifugation. The yield of the obtained **IMOF‐DMF** was 85% (calculated from H_3_L). The elemental contents (%) were analysed, and the theoretical values were: H, 3.54; N, 7.08; C, 48.43. The experimental values were: H, 3.55; N, 7.00; and C, 48.51. The infrared characteristic absorption peaks (KBr cm^−1^, Figure S1, Supporting Information) of **IMOF‐DMF** were: 3439 (m), 3009 (w), 2804 (s), 2475 (w). 1619 (s), 1440 (s), 1357 (s), 1118 (m), 841 (w), 775 (s), 726 (s), 614 (s), 535 (m), 424 (w).

### Synthesis of **IMOF‐DEF**

First, 14 mg of Zn(NO_3_)_2_·6H_2_O with 5.7 mg of H_3_L ligand was added to 4 mL of a mixed solvent composed of DEF and H_2_O in the ratio of 3:1 by volume and stirred thoroughly until a clear solution was formed. Subsequently, 10 µL of concentrated nitric acid was added to this solution, and the mixed solution was transferred to a 25 mL polytetrafluoroethylene autoclave reactor and heated at 120°C for 3 days. At the end of the reaction, the product was washed by using DI water, and finally transparent bulk crystals **IMOF‐DEF** were obtained. The yield of the obtained **IMOF‐DEF** was 90% (calculated from H_3_L). The elemental contents (%) were analysed, and the theoretical values were: H, 2.41; N, 6.74; C, 52.48. The experimental values were: H, 2.42; N, 6.79; and C, 52.01. The infrared characteristic absorption peaks (KBr cm^−1^, Figure S2, Supporting Information) of **IMOF‐DEF** were: 3439 (m), 2821 (m), 2728 (m), 2520 (w). 1607 (s), 1438 (m), 1344 (s), 1113 (m), 771 (m), 615 (m).

### Preparation of IMOF@PVA

First, 1 g of PVA was soaked in 20 mL of DI water for not less than 6 h. Subsequently, the temperature was slowly raised to 90°C and stirred slowly at this temperature until the PVA was completely dissolved. Next, 20 mg of **IMOF‐DMF** was added to the PVA solution, which was thoroughly mixed by ultrasound treatment, and the air bubbles in the solution were removed. After that, the suspension was injected into a custom‐made mould using a syringe, and the mould was frozen in a −20°C refrigerator for not less than 4 h. After removing the mould, it was defrosted at room temperature, again for not less than 4 h. The above process was repeated 4 to 5 times until IMOF@PVA formed a gel. Finally, the moulds were carefully removed to obtain the product in the target shape.

### Theoretical Calculations

Density‐functional theory (DFT) and time‐dependent DFT (TD‐DFT) calculations were carried out using the Gaussian 16W package in order to investigate the energy level structure of the singly‐ and triplet‐excited states. Based on the optimised ground state structure, the energies of the lowest singly excited state (S_1_) and triplet excited state (T_n_) are calculated using B3LYP/6‐31G(d). The fch files of the optimised structures were input into VMD 1.9.3 and Multiwfn 3.8 to plot the HOMO and LUMO orbitals.^[^ ^67^, ^68^ ^]^ To calculate the SOC between S_m_‐T_n_, PBE1PBE/def‐TZVP was chosen to optimise the ground state structure, and then the SOC was calculated using the ORCA 5.0 program B3LYP/def‐TZVP.

CCDC 2421368 and 2421367 contain the supplementary crystallographic data for this paper. These data were obtained free of charge from The Cambridge Crystallographic Data Centre via www.ccdc.cam.ac.uk/data_request/cif.

## Conflict of Interest

The authors declare no conflict of interest.

## Supporting information



Supporting Information

Supplemental Movie 1

Supplemental Movie 1

Supplemental Movie 1

Supplemental Movie 1

Supporting Information

## Data Availability

The data that support the findings of this study are available from the corresponding author upon reasonable request.
