# Supplementary material for: Molecular Engineering of Ionic Metal‐Organic Frameworks via Ligand Conjugation Modulation for Tailored Phosphorescence and Multilevel Encryption
Source: Adv Sci (Weinh). 2025 Jul 12;12(39):e09013. doi: 10.1002/advs.202509013 (PMC12533150; doi:10.1002/advs.202509013)
Supplement: Supplementary file 1 — Supporting Information [file ADVS-12-e09013-s006.docx]

Supporting Information

Molecular Engineering of Ionic Metal-Organic Frameworks via Ligand Conjugation Modulation for Tailored Phosphorescence and Multilevel Encryption

Qi Wu^[a]^, Xinyu Gao^[a]^, Wenyan Zhang*^[a]^, Yaoyu Wang^[a]^

**Table S1.** Crystallographic data for **IMOF-DMF** and **IMOF-DEF**

| Complex | **IMOF-DMF** | **IMOF-DEF** |
| --- | --- | --- |
| Formula | C_32_H_28_Zn_2_N_4_O_12_ | C_18_H_10_ZnN_2_O_6_ |
| CCDC | 2421368 | 2421367 |
| Mr | 791.32 | 415.67 |
| Crystal system | Orthorhombic | Orthorhombic |
| Space group | *P*2_1_2_1_2 | *Pbcn* |
| *a* [Å] | 13.668(3) | 13.754(11) |
| *b* [Å] | 15.788(7) | 15.899(13) |
| *c* [Å] | 16.434(4) | 16.28(2) |
| *α* [°] | 90 | 90 |
| *β* [°] | 90 | 90 |
| *γ* [°] | 90 | 90 |
| *V* [Å^3^] | 3546(2) | 3558(6) |
| *Z* | 4 | 8 |
| D_calcd_. [g·cm^-3^] | 1.480 | 1.582 |
| *F* [000] | 1612.0 | 1744.0 |
| GOF on *F*^2^ | 1.027 | 1.068 |
| *R*_1_^a^ [I > 2σ(I)] | 0.0427 | 0.0340 |
| w*R*_2_^b^ (all data) | 0.1064 | 0.0877 |
| ^a^R_1_ = ∑\|\|*F_o_*\|-\|*F_c_*\|\|/∑\|*F_o_*\|. ^b^*wR_2_*=[∑*w*(*F_o_*^2^- *F_c_*^2^)^2^/∑*w*(*F_o_*^2^)^2^]^1/2^ | | |

**Table S2.** Bond lengths [Å] and angles [deg] of **IMOF-DMF**

| **IMOF-DMF** |  |  |  |
| --- | --- | --- | --- |
| Zn(1)-O(2)#1 | 1.976(5) | O(2)#1-Zn(1)-N(1)#2 | 96.2(2) |
| Zn(1)-O(4) | 1.925(5) | O(4)-Zn(1)-O(2)#1 | 103.4(2) |
| Zn(1)-O(7) | 1.964(5) | O(4)-Zn(1)-O(7) | 96.8(2) |
| Zn(1)-N(1)#2 | 2.036(6) | O(4)-Zn(1)-N(1)#2 | 114.2(2) |
| Zn(2)-O(11)#3 | 1.995(5) | O(7)-Zn(1)-O(2)#1 | 124.1(2) |
| Zn(2)-O(5)#3 | 1.945(5) | O(7)-Zn(1)-N(1)#2 | 121.6(2) |
| Zn(2)-O(9) | 1.961(4) | O(11)#3-Zn(2)-O(5)#4 | 96.5(4) |
| Zn(2)-N(3)#5 | 2.056(11) | O(11)#3-Zn(2)-N(3)#5 | 108.4(2) |
| O(6)-C(10) | 1.221(9) | O(5)3-Zn(2)-N(2)#5 | 125.5(2) |
| O(11)-C(23) | 1.239(8) | O(5)3-Zn(2)-O(11)#4 | 104.8(2) |
| O(5)-C(10) | 1.305(9) | O(9)-Zn(2)-O(11)# | 104.8(2) |
| O(2)-C(5) | 1.231(9) | O(9)-Zn(2)-N(2)#5 | 100.8(2) |
| O(4)-C(13) | 1.304(9) | C(23)-O(11)-Zn(2)# | 124.4(5) |

Symmetry transformations used to generate equivalent atoms：#1:1/2-X,-1/2+Y,1-Z; #2:1-X,1-Y,+Z; #3:1/2-X,-1/2+Y,2-Z; #4:1/2-X,1/2+Y,2-Z; #5:-1/2+X,1/2-Y,2-Z.

**Table S3.** Bond lengths [Å] and angles [deg] of **IMOF-DEF**

| **IMOF-DMF** |  |  |  |
| --- | --- | --- | --- |
| Zn(1)-O(1)#1 | 2.012(3) | O(1)#1-Zn(1)-N(1)#2 | 111.18(9) |
| Zn(1)-O(4)#2 | 1.917(2) | O(4)#3-Zn(1)-O(1)#1 | 97.35(9) |
| Zn(1)-O(5) | 1.952(3) | O(4)#3-Zn(1)-O(5) | 120.45(9) |
| Zn(1)-N(1)#3 | 2.022(3) | O(4)#3-Zn(1)-N(1)#2 | 121.42(11) |
| O(1)-C(1) | 1.264(3) | O(5)-Zn(1)-O(1)#1 | 104.90(9) |
| O(2)-C(1) | 1.221(3) | O(5)-Zn(1)-N(1)#2 | 100.58(10) |
| O(3)-C(5) | 1.245(4) | C(1)-O(1)-Zn(1)#4 | 126.90(19) |
| O(4)-C(5) | 1.297(4) | C(5)-O(4)-Zn(1)#5 | 111.92(18) |
| N(1)-C(9) | 1.358(4) | C(14)-O(5)-Zn(1) | 124.8(2) |
| N(1)-C(10) | 1.341() | C(9)-N(1)-Zn(1)#6 | 123.92(19) |
| N(2)-C(16) | 1.406(17) | C(10)-N(1)-Zn(1)#6 | 114.9(2) |

Symmetry transformations used to generate equivalent atoms：#1：3/2-X,-1/2+Y,+Z; #2：3/2-X,1/2-Y,1/2+Z; #3：1/2+X,1/2-Y,1-Z; #4：3/2-X,1/2+Y,+Z; #5:3/2-X,1/2-Y,-1/2+Z; #6:-1/2+X,1/2-Y,1-Z

**Table S4.** Phosphorescence characteristics of IMOF-DMF compared with typical MOFs in literature^[S1-S9]^.

| **MOFs** | **Afterglow/s** | **Lifetime/ms** | **Quantum yield** | **Ref.** |
| --- | --- | --- | --- | --- |
| SUST-WJ-12 | 2.5 | 69.92 | 4.9% | S1 |
| SUST-WJ-13 | 2.5 | 63.22 | 40% |  |
| Cd-DMF | 5 | 187 | 6.28% | S2 |
| CAU-10-H | 2.5 | 638 | 25.2% | S3 |
| CAU-10-OCH_3_ | 0.5 | 101 | 4.4% |  |
| ZnSr-H_2_O | 7.0 | 195.4 | 6.8% | S4 |
| ZnBa-H_2_O | 7.5 | 162.5 | 9.0% |  |
| Zn-DCPS-BIMB | 3.5 | 451.6 | 1.32% | S5 |
| A2 | 0.002 | 1.6 | 2.5% | S6 |
| A3 | 0.012 | 3.35 | 6.51% |  |
| NKU-132-95% | 0.5 | 30.88 | 0.94% | S7 |
| NKU-132-80%DMA | 1 | 6.518 | 1.25% |  |
| NKU-132-70%DEA | 3 | 121.3 | 1.45% |  |
| 1 | 2.5 | 264.6 | 11.94% | S8 |
| 2 | <0.5 | 83.0 | 5.77% |  |
| 1-DMF | 4 | 472 | 4.76% | S9 |
| **IMOF-DMF** | 3 | 206 | 12.27% | **This Work** |
| **IMOF-DEF** | 0.8 | 173 | 1.76% |  |


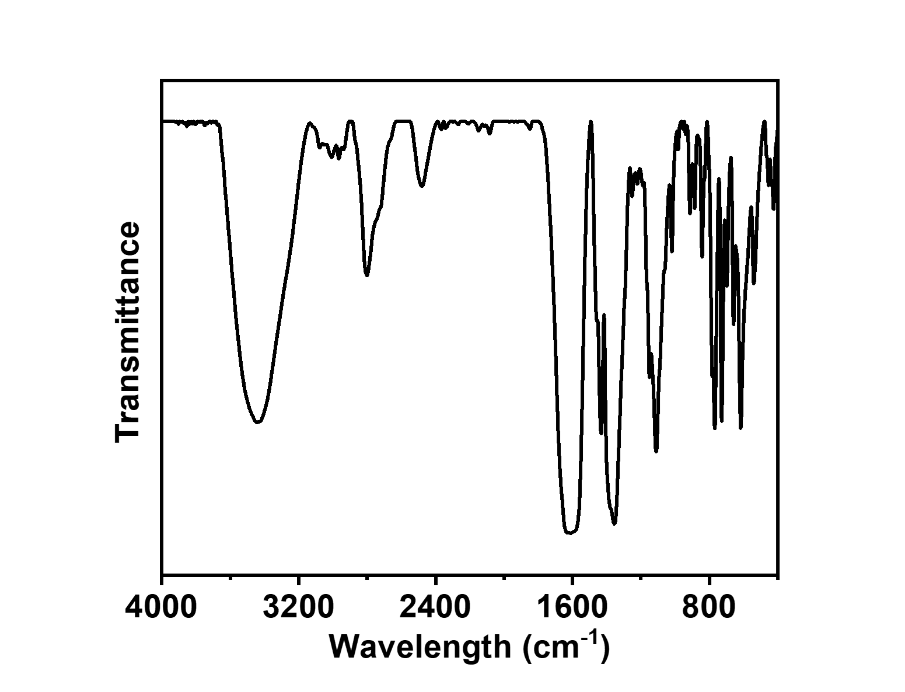


**Figure S1** FTIR spectra for **IMOF-DMF**.


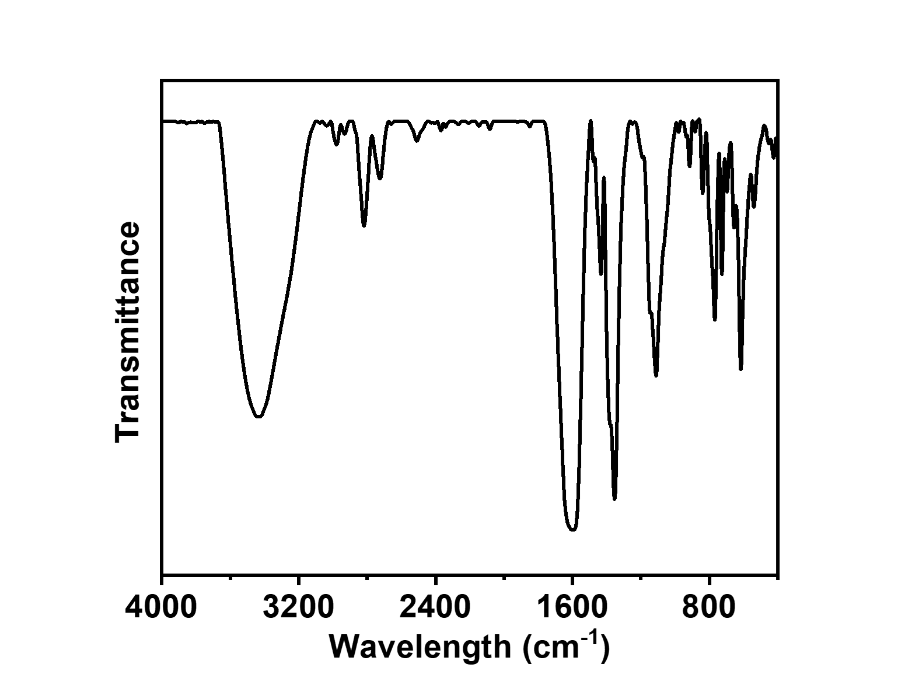


**Figure S2** FTIR spectra for **IMOF-DEF**.

**
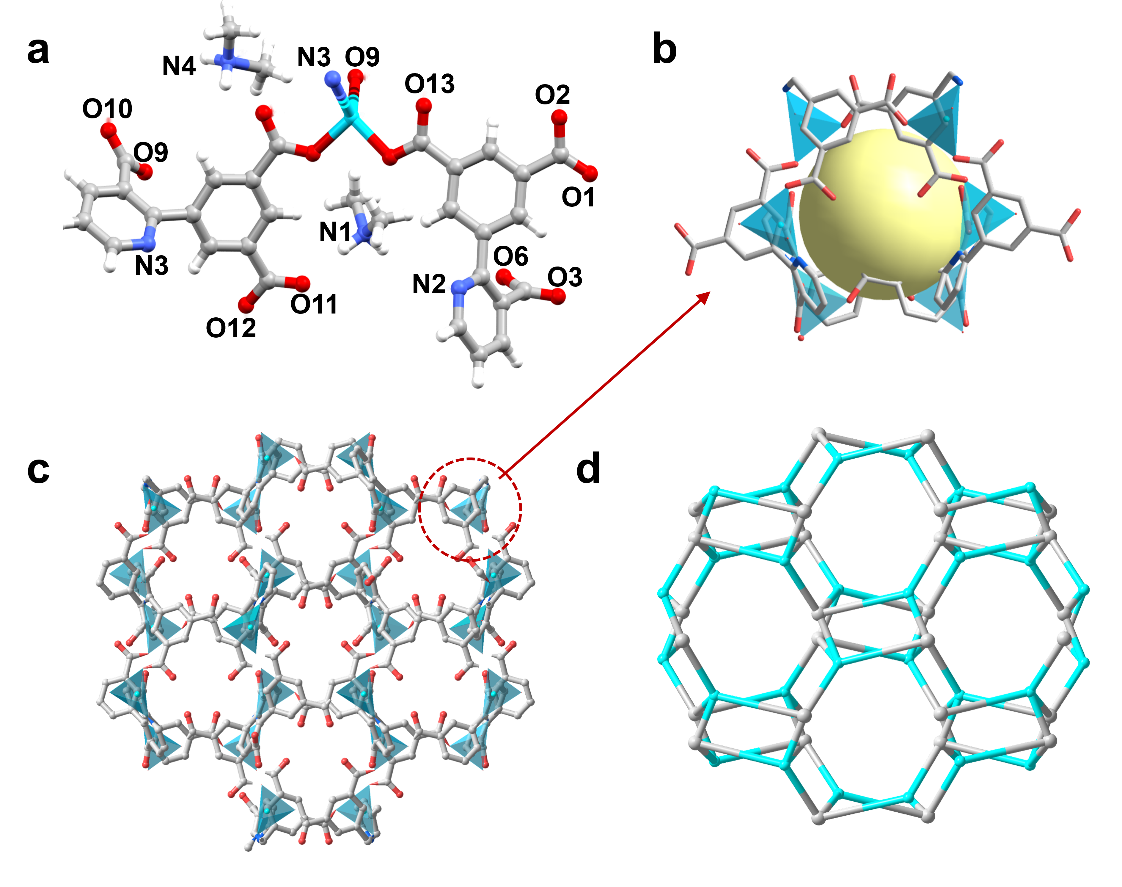
**

**Figure S3** a) Coordination environment diagrams of **IMOF-DMF**; b) and c) are the channel environment diagrams of **IMOF-DMF** and **IMOF-DEF** (guest ions are deleted) and partial enlarged diagrams; d) topological structure diagrams of **IMOF-DMF** and **IMOF-DEF**.


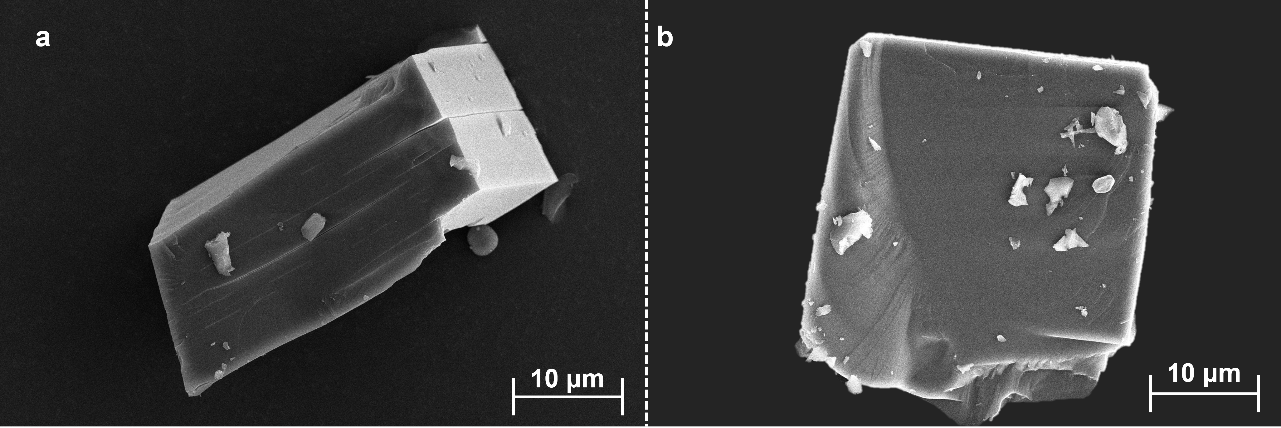


**Figure S4** The SEM pictures of a) IMOF-DMF and b) IMOF-DEF, respectively.

**
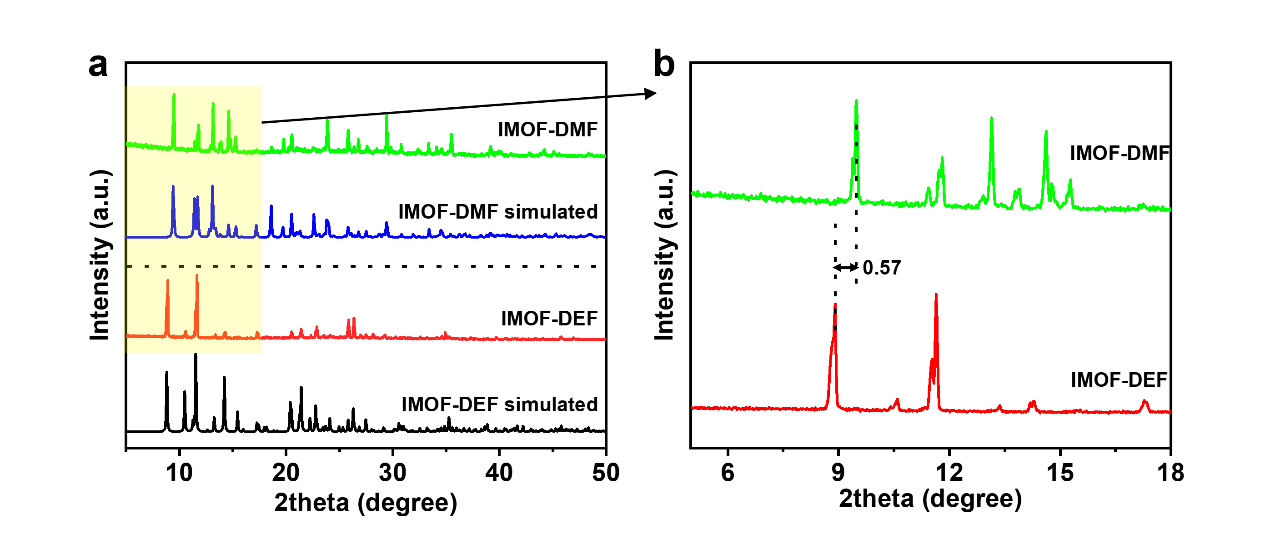
**

**Figure S5** a) Simulated PXRD data of single-crystal structure of **IMOFs** vs. test data; b) partial enlarged diagrams.

**
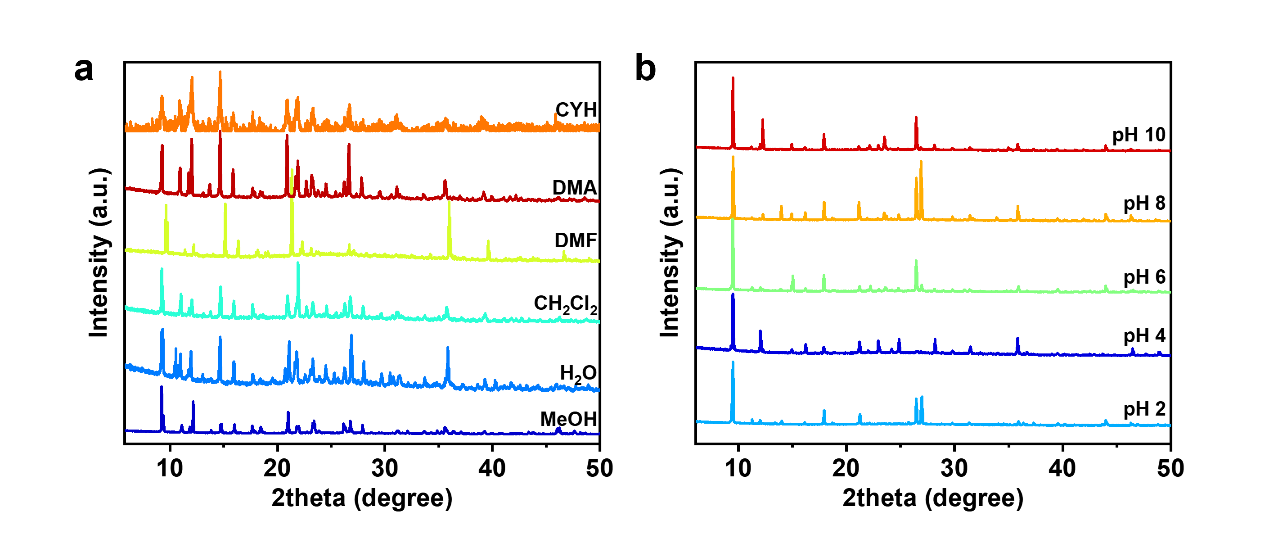
**

**Figure S6** PXRD patterns of **IMOF-DMF** tested after immersion in a) different solvents and b) different pH aqueous solutions.

**
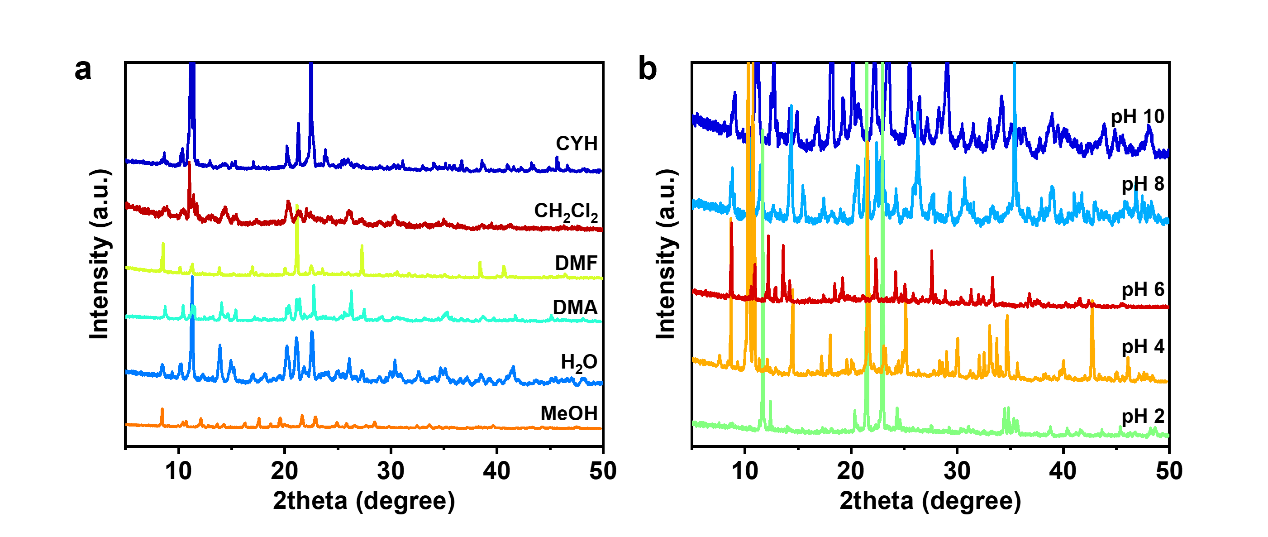
**

**Figure S7** PXRD patterns of **IMOF-DEF** tested after immersion in a) different solvents and b) different pH aqueous solutions.


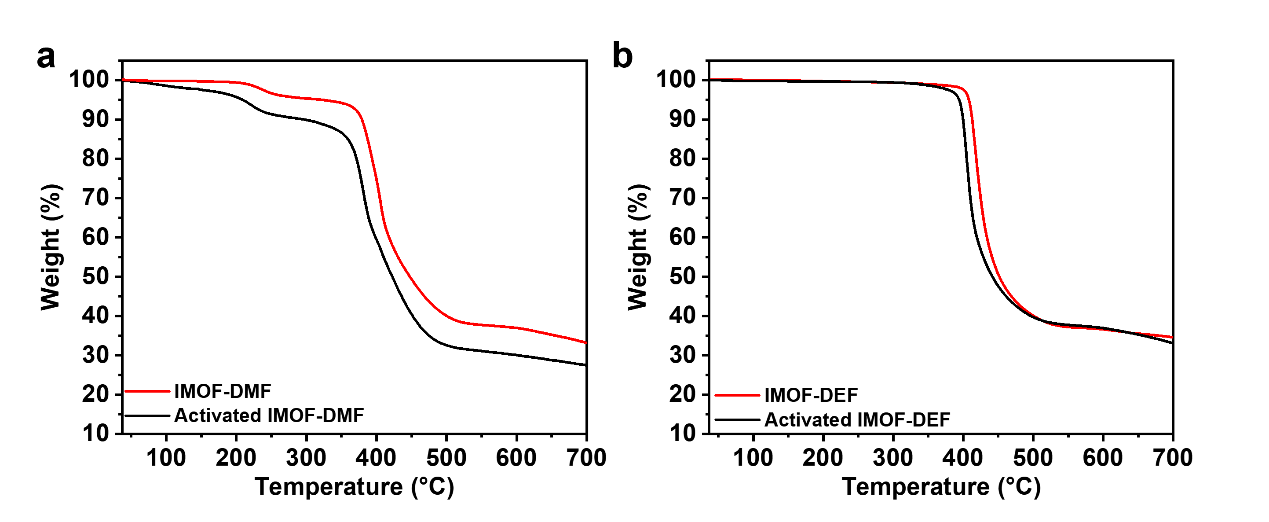


**Figure S8** TGA curves of a) **IMOF-DMF** and b) **IMOF-DEF** and their activated materials, respectively.

**
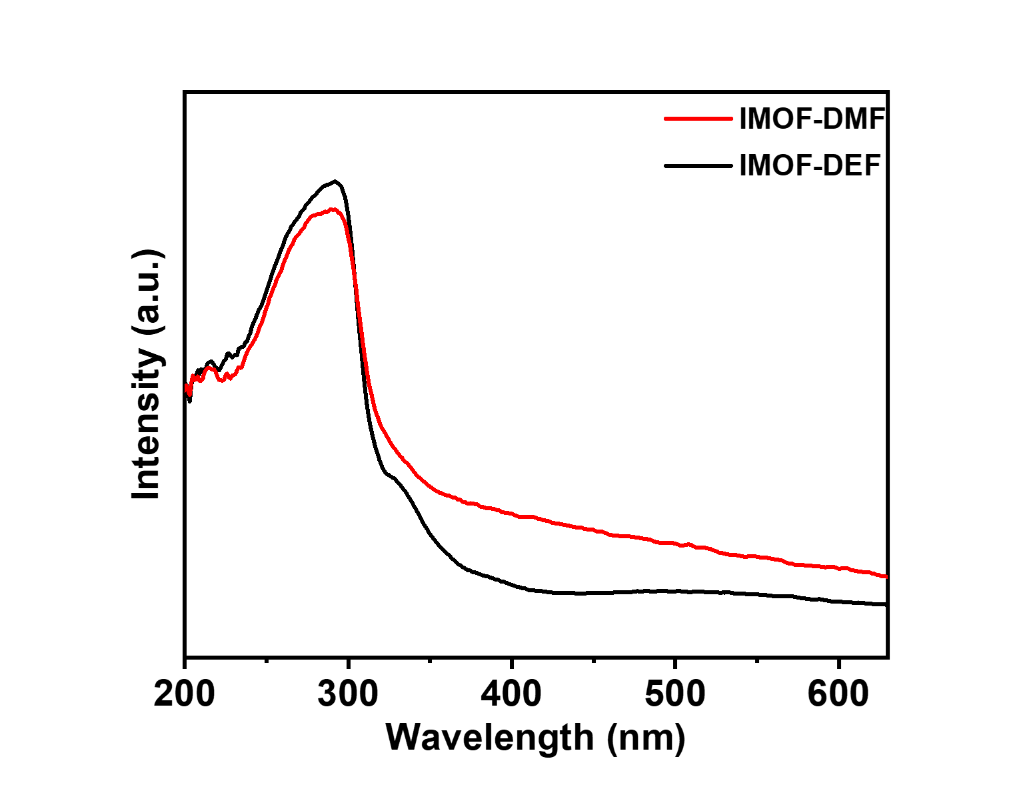
**

**Figure S9** UV-Vis DRS curves of **IMOF-DMF** and **IMOF-DEF.**


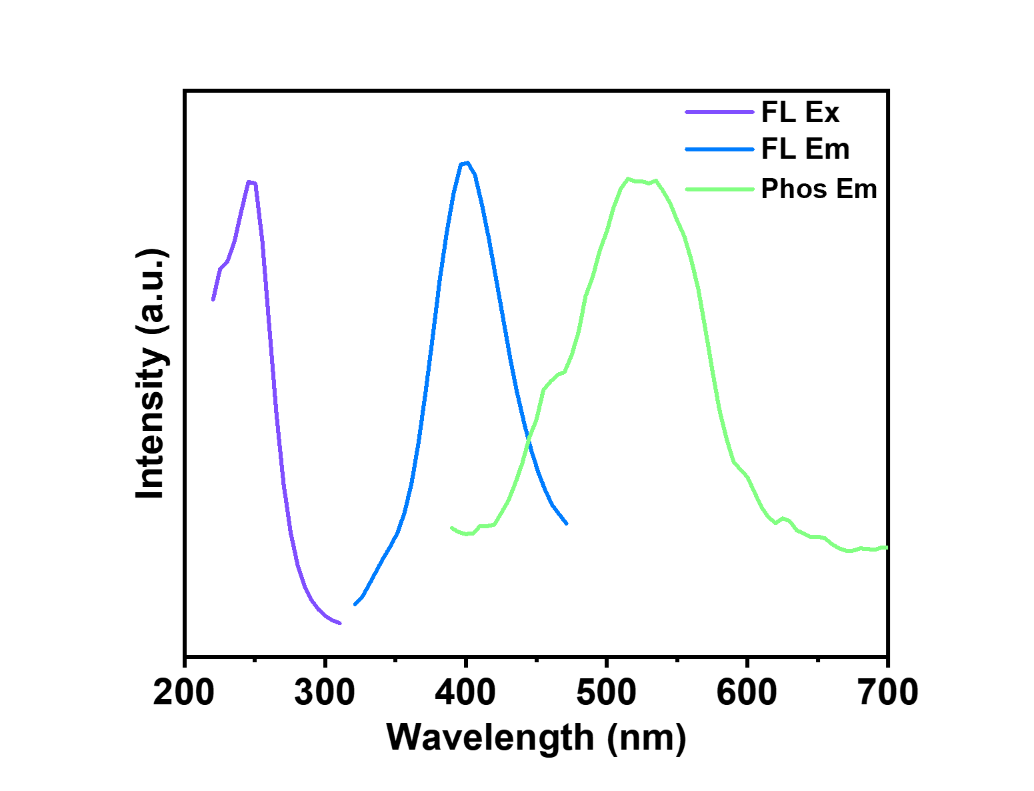


**Figure S10** PL spectra of H_3_L


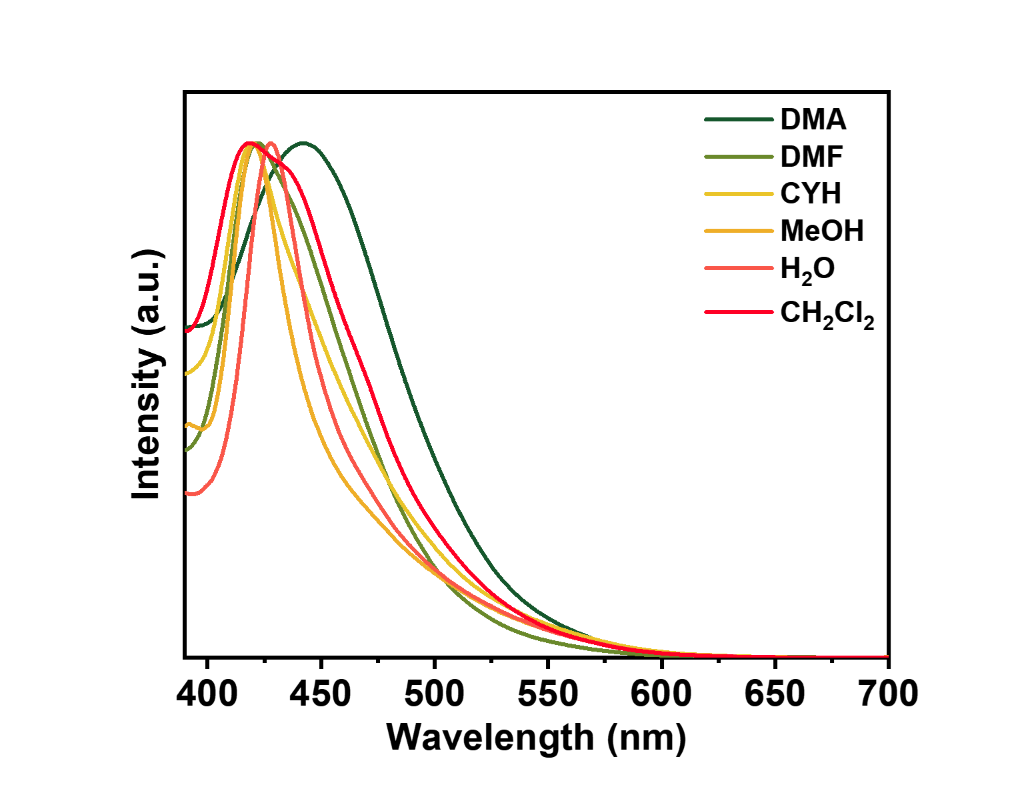


**Figure S11** PL spectra of **IMOF-DMF** in different organic solvents.


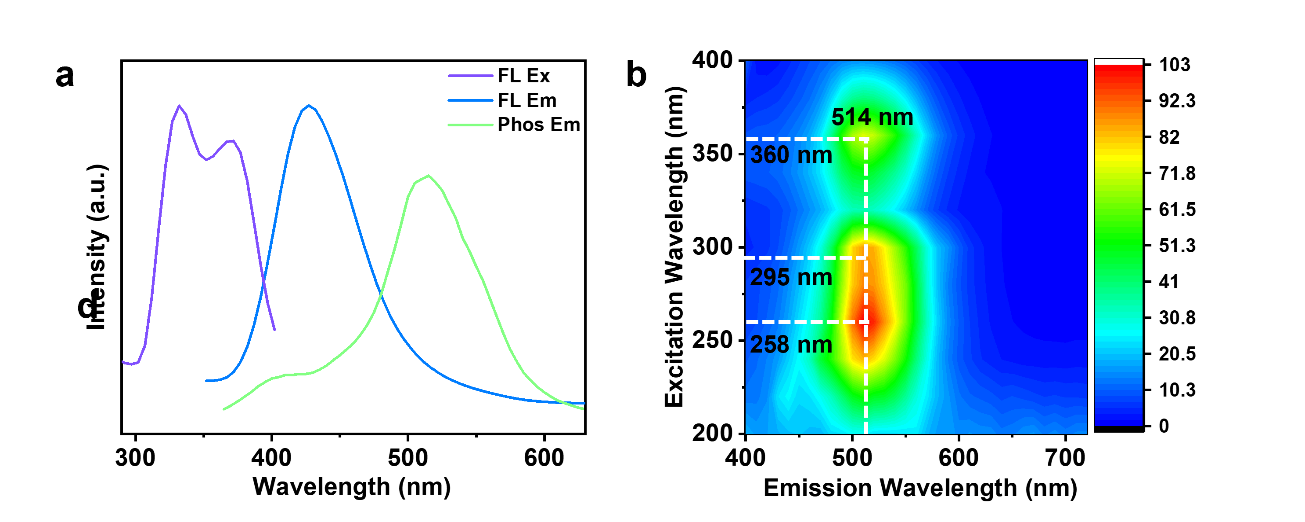


**Figure S12** PL spectra of **IMOF-DEF**.


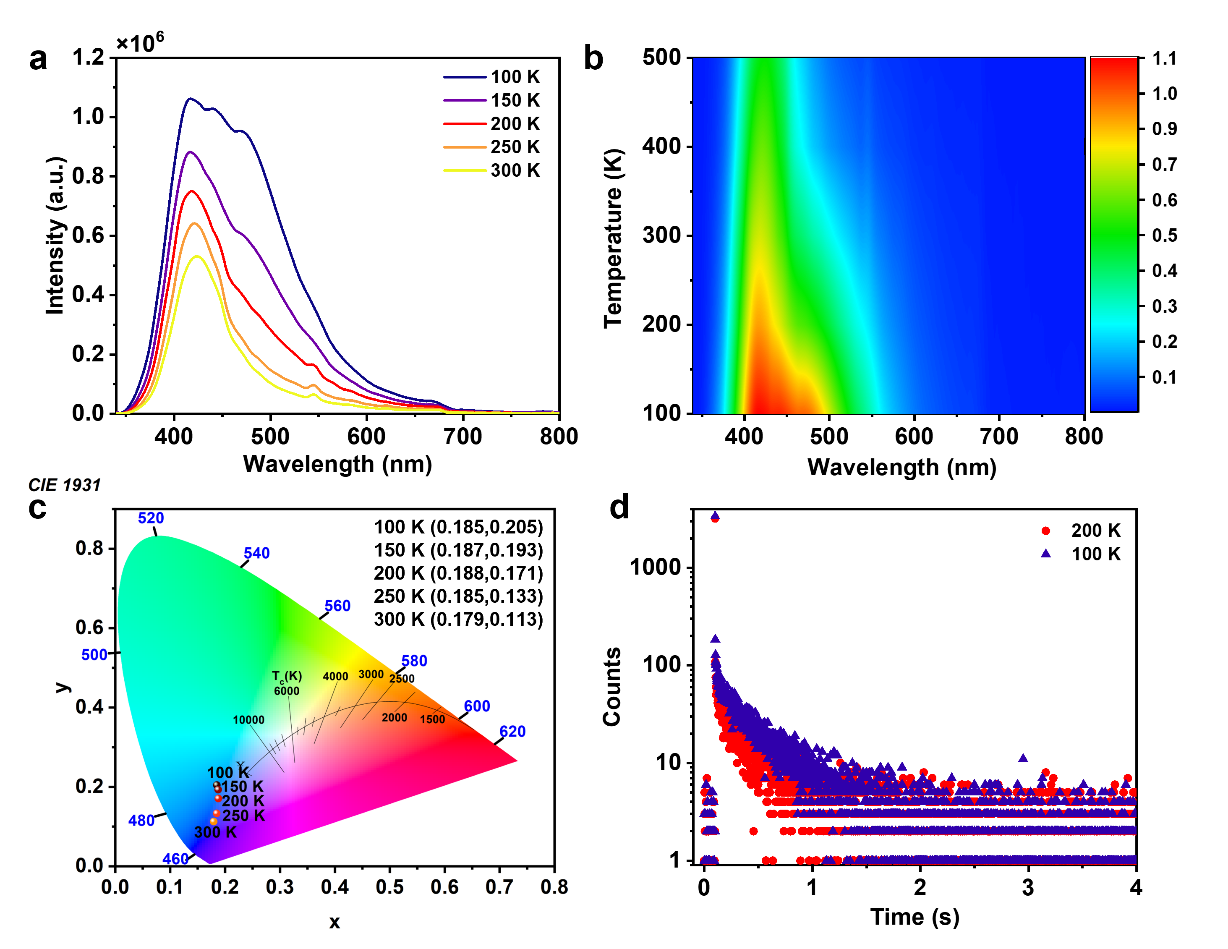


**Figure S13** PL spectra of **IMOF-DMF**. a) Typical curves; b) 2D contours, and c) corresponding CIE coordinates for the temperature-dependent emission of **IMOF-DMF** at the excitation wavelength of 328 nm; d) Phosphorescence decay curves of **IMOF-DMF** at 100 and 200 K.


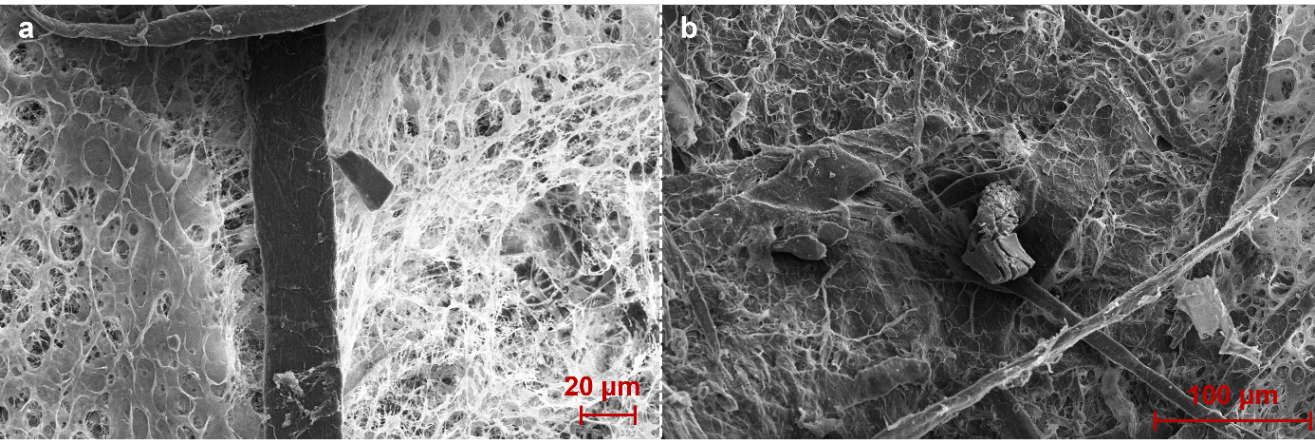


**Figure S14** The SEM pictures of IMOF@PVA.

**Reference**

1. Z. Wang, J. J. Liu, M. Y. Li, G. *Chen, Chem. Eng. J.* 2023, 462, 142154.
2. R. Feng, Z. Y. Li, Z. Q. Yao, Z. Guo, Y. N. Zhang, H. X. Sun, W. Li, X. H. Bu, *Sci.* *China Chem*. 2022, 65, 128-134.
3. Q. Yu, J. Zhang, J. W. Y. Lam, D. Yang, J. Sun, B. Z. Tang, *ACS Mater. Lett*. 2023, 5, 2691-2699.
4. S. Liu, Y. Lin, D. Yan, *Sci. China Chem*. 2023, 66, 3532-3538.
5. J. Xue, Z. Qi, D. Yan, G.-P. Yang, Y.-Y. Wang, *Angew. Chem. Int. Ed*. 2025, 64 e202501951.
6. L. Geng, K. Wang, R. Sun, C. T. Li, X. R. Li, M. Zhang, M. H. Yu, Z. Chang, X. H. Bu, *CCS Chem*. 2025, 7, 416-428.
7. S. Li, Y. Lin, D. Yan, *Chin. Chem. Lett*. 2023, 34, 107952.
8. W. Q. Zhang, B. L. Zhang, T. Wang, J. Chen, Z. Y. Li, R. H. Wang, S. Liu, J. J. Zhang, *J. Mater. Chem. A* 2024, 12, 7732-7741.
9. X. Yang, D. Yan, *Chem. Sci.* 2016, 7, 4519-4526.
