# Supplementary material for: Molecular Engineering of Ionic Metal‐Organic Frameworks via Ligand Conjugation Modulation for Tailored Phosphorescence and Multilevel Encryption
Source: Adv Sci (Weinh). 2025 Jul 12;12(39):e09013. doi: 10.1002/advs.202509013 (PMC12533150; doi:10.1002/advs.202509013)

checkCIF/PLATON report

Structure factors have been supplied for datablock(s) mo_13wq0830_0m_a

THIS REPORT IS FOR GUIDANCE ONLY. IF USED AS PART OF A REVIEW PROCEDURE FOR PUBLICATION, IT SHOULD NOT REPLACE THE EXPERTISE OF AN EXPERIENCED CRYSTALLOGRAPHIC REFEREE.

No syntax errors found.   CIF dictionary   Interpreting this report

Datablock: mo_13wq0830_0m_a

```
Bond precision:   C-C = 0.0042 A          Wavelength=0.71073

Cell:              a=13.754(11)      b=15.889(13)      c=16.28(2)
                   alpha=90          beta=90           gamma=90
Temperature:       240 K

                Calculated                   Reported
Volume          3558(6)                      3557(6)
Space group     P b c n                      P b c n
Hall group      -P 2n 2ab                    -P 2n 2ab
Moiety formula  C14 H6 N O6 Zn, C4 H12 N     C14 H6 N O6 Zn, C4 H12 N
Sum formula     C18 H18 N2 O6 Zn             C18 H18 N2 O6 Zn
Mr              423.73                       423.71
Dx,g cm-3       1.582                        1.582
Z               8                            8
Mu (mm-1)       1.419                        1.420
F000            1744.0                       1744.0
F000'           1747.04
h,k,lmax        16,19,20                     16,19,19
Nref            3464                         3238
Tmin,Tmax       0.808,0.843                  0.650,0.745
Tmin'           0.808

Correction method= # Reported T Limits: Tmin=0.650 Tmax=0.745
AbsCorr = MULTI-SCAN

Data completeness= 0.935          Theta(max)= 25.905

                                                    wR2(reflections)=
R(reflections)= 0.0340( 2627)                       0.0877( 3238)

S = 1.067                  Npar= 304
```

The following ALERTS were generated. Each ALERT has the format
test-name_ALERT_alert-type_alert-level.
Click on the hyperlinks for more details of the test.

Alert level C

```
PLAT148_ALERT_3_C s.u. on the      c    - Axis is (Too) Large ....      0.020 Ang.
PLAT369_ALERT_2_C Long   C(sp2)-C(sp2) Bond  C1       - C2       .       1.53 Ang.
PLAT911_ALERT_3_C Missing FCF Refl Between Thmin & STh/L=    0.600         51 Report
                4  0  0,   0  2  0,  11 13  0,  10 14  0,   0  0  2,   0  0  4,
               14  2 10,  13  0 12,  11  8 12,  12  2 13,  12  3 13,  11  6 13,
               11  3 14,   9  8 14,  10  3 15,   9  6 15,   7  9 15,   6 10 15,
                9  1 16,   9  2 16,   9  3 16,   8  5 16,   7  7 16,   6  8 16,
                5  9 16,   8  1 17,   7  4 17,   6  6 17,   5  7 17,   4  8 17,
                1  9 17,   2  9 17,   6  0 18,   6  1 18,   6  2 18,   5  3 18,
                5  4 18,   4  5 18,   3  6 18,   1  7 18,   2  7 18,   2  1 19,
                3  1 19,   0  2 19,   1  2 19,   2  2 19,   3  2 19,   1  3 19,
                2  3 19,   0  4 19,   1  4 19,
```

Alert level G

```
PLAT002_ALERT_2_G Number of Distance or Angle Restraints on AtSite         14 Note
PLAT003_ALERT_2_G Number of Uiso or U(i,j)  Restrained non-H-Atoms         14 Report
PLAT004_ALERT_5_G Polymeric Structure Found with Maximum Dimension          3 Info
PLAT007_ALERT_5_G Number of Unrefined Donor-H Atoms .............           4 Report
              H2AA  H2AB  H2A   H2B
PLAT176_ALERT_4_G The CIF-Embedded .res File Contains SADI Records          9 Report
PLAT178_ALERT_4_G The CIF-Embedded .res File Contains SIMU Records          2 Report
PLAT188_ALERT_3_G A Non-default SIMU Restraint Value has been used     0.0100 Report
PLAT188_ALERT_3_G A Non-default SIMU Restraint Value has been used     0.0100 Report
PLAT191_ALERT_3_G A Non-default SADI Restraint Value has been used     0.0400 Report
PLAT191_ALERT_3_G A Non-default SADI Restraint Value has been used     0.0400 Report
PLAT191_ALERT_3_G A Non-default SADI Restraint Value has been used     0.0400 Report
PLAT191_ALERT_3_G A Non-default SADI Restraint Value has been used     0.0400 Report
PLAT191_ALERT_3_G A Non-default SADI Restraint Value has been used     0.0400 Report
PLAT301_ALERT_3_G Main Residue  Disorder .............(Resd    1)          5% Note
PLAT302_ALERT_4_G Anion/Solvent/Minor-Residue Disorder (Resd    2)       100% Note
PLAT302_ALERT_4_G Anion/Solvent/Minor-Residue Disorder (Resd    3)       100% Note
PLAT720_ALERT_4_G Number of Unusual/Non-Standard Labels .........           2 Note
              H2AA    H2AB
PLAT773_ALERT_2_G Check long C-C Bond in CIF: C15      --C16             1.71 Ang.
PLAT860_ALERT_3_G Number of Least-Squares Restraints .............        153 Note
PLAT910_ALERT_3_G Missing # of FCF Reflection(s) Below Theta(Min).          1 Note
                1  1  0,
PLAT912_ALERT_4_G Missing # of FCF Reflections Above STh/L=  0.600        175 Note
PLAT913_ALERT_3_G Missing # of Very Strong Reflections in FCF ....          3 Note
                4  0  0,   0  0  2,   0  0  4,
PLAT933_ALERT_2_G Number of HKL-OMIT Records in Embedded .res File          1 Note
                1  1  0,
PLAT969_ALERT_5_G The 'Henn et al.' R-Factor-gap value ..........       3.710 Note
              Predicted wR2: Based on SigI**2  2.36 or SHELX Weight  8.22
PLAT978_ALERT_2_G Number C-C Bonds with Positive Residual Density.          8 Info
```

0 **ALERT level A** = Most likely a serious problem - resolve or explain
0 **ALERT level B** = A potentially serious problem, consider carefully

```
 3 ALERT level C = Check. Ensure it is not caused by an omission or oversight
25 ALERT level G = General information/check it is not something unexpected

 0 ALERT type 1 CIF construction/syntax error, inconsistent or missing data
 6 ALERT type 2 Indicator that the structure model may be wrong or deficient
13 ALERT type 3 Indicator that the structure quality may be low
 6 ALERT type 4 Improvement, methodology, query or suggestion
 3 ALERT type 5 Informative message, check
```

It is advisable to attempt to resolve as many as possible of the alerts in all categories. Often the minor alerts point to easily fixed oversights, errors and omissions in your CIF or refinement strategy, so attention to these fine details can be worthwhile. In order to resolve some of the more serious problems it may be necessary to carry out additional measurements or structure refinements. However, the purpose of your study may justify the reported deviations and the more serious of these should normally be commented upon in the discussion or experimental section of a paper or in the "special_details" fields of the CIF. checkCIF was carefully designed to identify outliers and unusual parameters, but every test has its limitations and alerts that are not important in a particular case may appear. Conversely, the absence of alerts does not guarantee there are no aspects of the results needing attention. It is up to the individual to critically assess their own results and, if necessary, seek expert advice.

Publication of your CIF in IUCr journals

A basic structural check has been run on your CIF. These basic checks will be run on all CIFs submitted for publication in IUCr journals (*Acta Crystallographica*, *Journal of Applied Crystallography*, *Journal of Synchrotron Radiation*); however, if you intend to submit to *Acta Crystallographica Section C* or *E* or *IUCrData*, you should make sure that full publication checks are run on the final version of your CIF prior to submission.

Publication of your CIF in other journals

Please refer to the *Notes for Authors* of the relevant journal for any special instructions relating to CIF submission.

PLATON version of 02/02/2025; check.def file version of 02/02/2025

Datablock mo_13wq0830_0m_a - ellipsoid plot

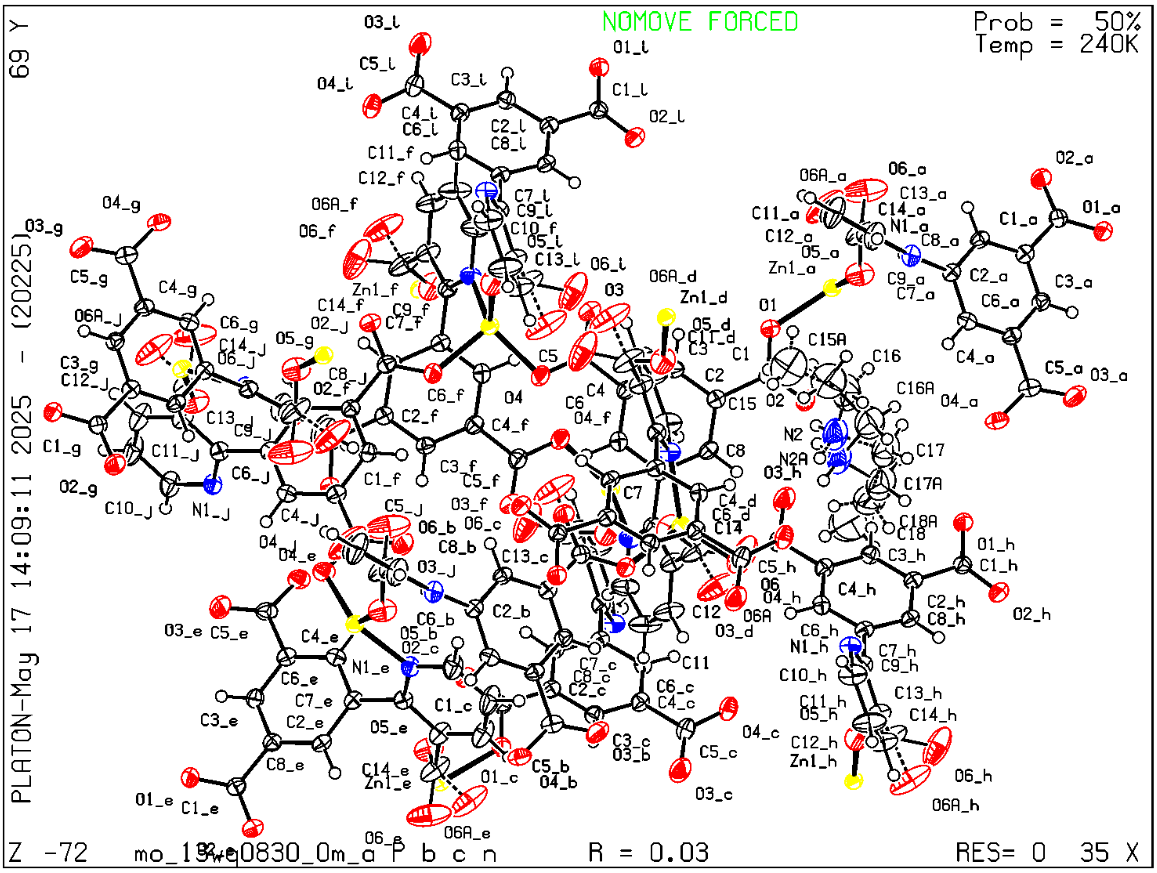

Supplement: Supplementary file 6 — Supporting Information [file ADVS-12-e09013-s002.zip › IMOF-DEF.pdf]
