# Supplementary material for: Molecular Engineering of Ionic Metal‐Organic Frameworks via Ligand Conjugation Modulation for Tailored Phosphorescence and Multilevel Encryption
Source: Adv Sci (Weinh). 2025 Jul 12;12(39):e09013. doi: 10.1002/advs.202509013 (PMC12533150; doi:10.1002/advs.202509013)

checkCIF/PLATON report

Structure factors have been supplied for datablock(s) a_b

THIS REPORT IS FOR GUIDANCE ONLY. IF USED AS PART OF A REVIEW PROCEDURE FOR PUBLICATION, IT SHOULD NOT REPLACE THE EXPERTISE OF AN EXPERIENCED CRYSTALLOGRAPHIC REFEREE.

No syntax errors found. CIF dictionary Interpreting this report

Datablock: a_b

Bond precision: C-C = 0.0104 A Wavelength=0.71073

Cell:	a=13.668(3)	b=15.788(7)	c=16.434(4)
	alpha=90	beta=90	gamma=90
Temperature:	200 K		

	Calculated	Reported
Volume	3546(2)	3546.1(18)
Space group	P 21 21 2	P 21 21 2
Hall group	P 2 2ab	P 2 2ab
Moiety formula	C28 H11 N2 O12 Zn2, C2 H8 N [+ solvent]	C28 H11 N2 O12 Zn2, C2 H8 N, 1[C2H8N]
Sum formula	C30 H19 N3 O12 Zn2 [+ solvent]	C32 H27 N4 O12 Zn2
Mr	744.26	790.31
Dx,g cm-3	1.394	1.480
Z	4	4
Mu (mm-1)	1.413	1.418
F000	1504.0	1612.0
F000'	1506.99	
h,k,lmax	16,19,19	16,18,19
Nref	6470[3623]	6230
Tmin,Tmax	0.967,0.986	0.665,0.745
Tmin'	0.958	

Correction method= # Reported T Limits: Tmin=0.665 Tmax=0.745
AbsCorr = MULTI-SCAN

Data completeness= 1.72/0.96 Theta(max)= 25.321

R(reflections)= 0.0427(5106) wR2(reflections)= 0.1064(6230)

S = 1.027 Npar= 436

The following ALERTS were generated. Each ALERT has the format
test-name_ALERT_alert-type_alert-level.
Click on the hyperlinks for more details of the test.

Alert level C

```
STRVA01_ALERT_4_C           Flack test results are ambiguous.
           From the CIF: _refine_ls_abs_structure_Flack    0.450
           From the CIF: _refine_ls_abs_structure_Flack_su    0.020
PLAT213_ALERT_2_C Atom O8              has ADP max/min Ratio .....        3.2 prolat
PLAT220_ALERT_2_C NonSolvent   Resd 1  C   Ueq(max)/Ueq(min) Range        3.1 Ratio
PLAT234_ALERT_4_C Large Hirshfeld Difference O10      --C32       .      0.16 Ang.
PLAT244_ALERT_4_C Low    'Solvent' Ueq as Compared to Neighbors of         N3 Check
PLAT260_ALERT_2_C Large Average Ueq of Residue Including        N3      0.102 Check
PLAT309_ALERT_2_C Single Bonded Oxygen (C-O > 1.3 Ang) ..........         O1A Check
PLAT341_ALERT_3_C Low Bond Precision on  C-C Bonds ...............    0.01039 Ang.
PLAT369_ALERT_2_C Long   C(sp2)-C(sp2) Bond  C17      - C18       .      1.53 Ang.
PLAT369_ALERT_2_C Long   C(sp2)-C(sp2) Bond  C22      - C23       .      1.53 Ang.
PLAT911_ALERT_3_C Missing FCF Refl Between Thmin & STh/L=    0.600         35 Report
                2  0  0,   4  0  0,  16  0  0,  16  1  0,   0  2  0,   0  4  0,
               10  9  0,  12 12  0,   3 13  0,  10 14  0,   0 18  0,   5 18  0,
                1  1  1,   0  2  1,   1  2  1,   0  3  1,   1  3  1,  10 14  1,
                0  0  2,   1  0  2,   0  1  2,   1  1  2,   8 15  3,   9 14  4,
                0 16  4,   6 16  4,   0 14  5,   7 15  5,  13 10  6,   8 14  6,
                0 12  9,   0 11 10,   0 10 11,   0  7 13,   0 11 16,
```

Alert level G

```
FORMU01_ALERT_2_G  There is a discrepancy between the atom counts in the
            _chemical_formula_sum and the formula from the _atom_site* data.
            Atom count from _chemical_formula_sum:C32 H27 N4 O12 Zn2
            Atom count from the _atom_site data:  C30 H19 N3 O12 Zn2
CELLZ01_ALERT_1_G Difference between formula and atom_site contents detected.
CELLZ01_ALERT_1_G ALERT: Large difference may be due to a
            symmetry error - see SYMMG tests
           From the CIF: _cell_formula_units_Z    4
           From the CIF: _chemical_formula_sum  C32 H27 N4 O12 Zn2
           TEST: Compare cell contents of formula and atom_site data

           atom    Z*formula  cif sites diff
           C        128.00    120.00    8.00
           H        108.00     76.00   32.00
           N         16.00     12.00    4.00
           O         48.00     48.00    0.00
           Zn         8.00      8.00    0.00
PLAT002_ALERT_2_G Number of Distance or Angle Restraints on AtSite          3 Note
PLAT003_ALERT_2_G Number of Uiso or U(i,j)  Restrained non-H-Atoms          3 Report
PLAT004_ALERT_5_G Polymeric Structure Found with Maximum Dimension          3 Info
PLAT007_ALERT_5_G Number of Unrefined Donor-H Atoms .............           2 Report
              H3A   H3B
PLAT033_ALERT_4_G Flack x Value Deviates > 3.0 * Sigma from Zero .      0.450 Note
```

```
PLAT041_ALERT_1_G Calc. and Reported SumFormula    Strings  Differ     Please Check
              Calc: C30 H19 N3 O12 Zn2
              Rep.: C32 H27 N4 O12 Zn2
PLAT042_ALERT_1_G Calc. and Reported MoietyFormula Strings  Differ     Please Check
              Calc: C28 H11 N2 O12 Zn2, C2 H8 N
              Rep.: C28 H11 N2 O12 Zn2, C2 H8 N, 1[C2H8N]
PLAT111_ALERT_2_G ADDSYM Detects New (Pseudo) Centre of Symmetry .         91 %Fit
PLAT113_ALERT_2_G ADDSYM Suggests Possible Pseudo/New  Space Group       Pbcn Check
              WARNING: Disordered Atoms Excluded from Analysis
              Check Model Parameter Symmetry for Reflection Data Support
PLAT152_ALERT_1_G The Supplied and Calc. Volume s.u. Differ by ...          2 Units
PLAT176_ALERT_4_G The CIF-Embedded .res File Contains SADI Records          1 Report
PLAT178_ALERT_4_G The CIF-Embedded .res File Contains SIMU Records          1 Report
PLAT186_ALERT_4_G The CIF-Embedded .res File Contains ISOR Records          1 Report
PLAT188_ALERT_3_G A Non-default SIMU Restraint Value has been used     0.0100 Report
PLAT299_ALERT_4_G Atom Site Occupancy Constrained at ............         0.5 Check
              O1      O1A
PLAT301_ALERT_3_G Main Residue  Disorder .............(Resd    1)          2% Note
PLAT343_ALERT_2_G Unusual sp?      Angle Range in Main Residue for        C27 Check
PLAT606_ALERT_4_G Solvent Accessible VOID(S) in Structure ........          ! Info
PLAT794_ALERT_5_G Tentative Bond Valency for Zn2       (II)      .       1.86 Info
PLAT860_ALERT_3_G Number of Least-Squares Restraints ............          31 Note
PLAT868_ALERT_4_G ALERTS Due to the Use of _smtbx_masks Suppressed          ! Info
PLAT909_ALERT_3_G Percentage of I>2sig(I) Data at Theta(Max) Still        59% Note
PLAT910_ALERT_3_G Missing # of FCF Reflection(s) Below Theta(Min).          3 Note
                0  0  1,   1  0  1,   0  1  1,
PLAT913_ALERT_3_G Missing # of Very Strong Reflections in FCF ....          3 Note
                4  0  0,   0  2  0,   0  4  0,
PLAT933_ALERT_2_G Number of HKL-OMIT Records in Embedded .res File         13 Note
               -3  3  1,  -1  1  1,  -1  2  1,  -1  3  1,   0  1  1,   0  2  0,
                0  3  1,   1  0  1,   1  0  2,   1  1  3,   1  2  1,   1  3  1,
                2  0  0,
PLAT941_ALERT_3_G Average HKL Measurement Multiplicity ..........         3.0 Low
PLAT969_ALERT_5_G The 'Henn et al.' R-Factor-gap value ..........       2.453 Note
              Predicted wR2: Based on SigI**2  4.34 or SHELX Weight 10.37
PLAT978_ALERT_2_G Number C-C Bonds with Positive Residual Density.          2 Info
PLAT992_ALERT_5_G Repd & Actual _reflns_number_gt Values Differ by          2 Check
```

---

   0 **ALERT level A** = Most likely a serious problem - resolve or explain
   0 **ALERT level B** = A potentially serious problem, consider carefully
  11 **ALERT level C** = Check. Ensure it is not caused by an omission or oversight
  32 **ALERT level G** = General information/check it is not something unexpected

   5 ALERT type 1 CIF construction/syntax error, inconsistent or missing data
  14 ALERT type 2 Indicator that the structure model may be wrong or deficient
   9 ALERT type 3 Indicator that the structure quality may be low
  10 ALERT type 4 Improvement, methodology, query or suggestion
   5 ALERT type 5 Informative message, check

It is advisable to attempt to resolve as many as possible of the alerts in all categories. Often the minor alerts point to easily fixed oversights, errors and omissions in your CIF or refinement strategy, so attention to these fine details can be worthwhile. In order to resolve some of the more serious problems it may be necessary to carry out additional measurements or structure refinements. However, the purpose of your study may justify the reported deviations and the more serious of these should normally be commented upon in the discussion or experimental section of a paper or in the "special_details" fields of the CIF. checkCIF was carefully designed to identify outliers and unusual parameters, but every test has its limitations and alerts that are not important in a particular case may appear. Conversely, the absence of alerts does not guarantee there are no aspects of the results needing attention. It is up to the individual to critically assess their own results and, if necessary, seek expert advice.

Publication of your CIF in IUCr journals

A basic structural check has been run on your CIF. These basic checks will be run on all CIFs submitted for publication in IUCr journals (*Acta Crystallographica*, *Journal of Applied Crystallography*, *Journal of Synchrotron Radiation*); however, if you intend to submit to *Acta Crystallographica Section C* or *E* or *IUCrData*, you should make sure that full publication checks are run on the final version of your CIF prior to submission.

Publication of your CIF in other journals

Please refer to the *Notes for Authors* of the relevant journal for any special instructions relating to CIF submission.

PLATON version of 02/02/2025; check.def file version of 02/02/2025

Datablock a_b - ellipsoid plot

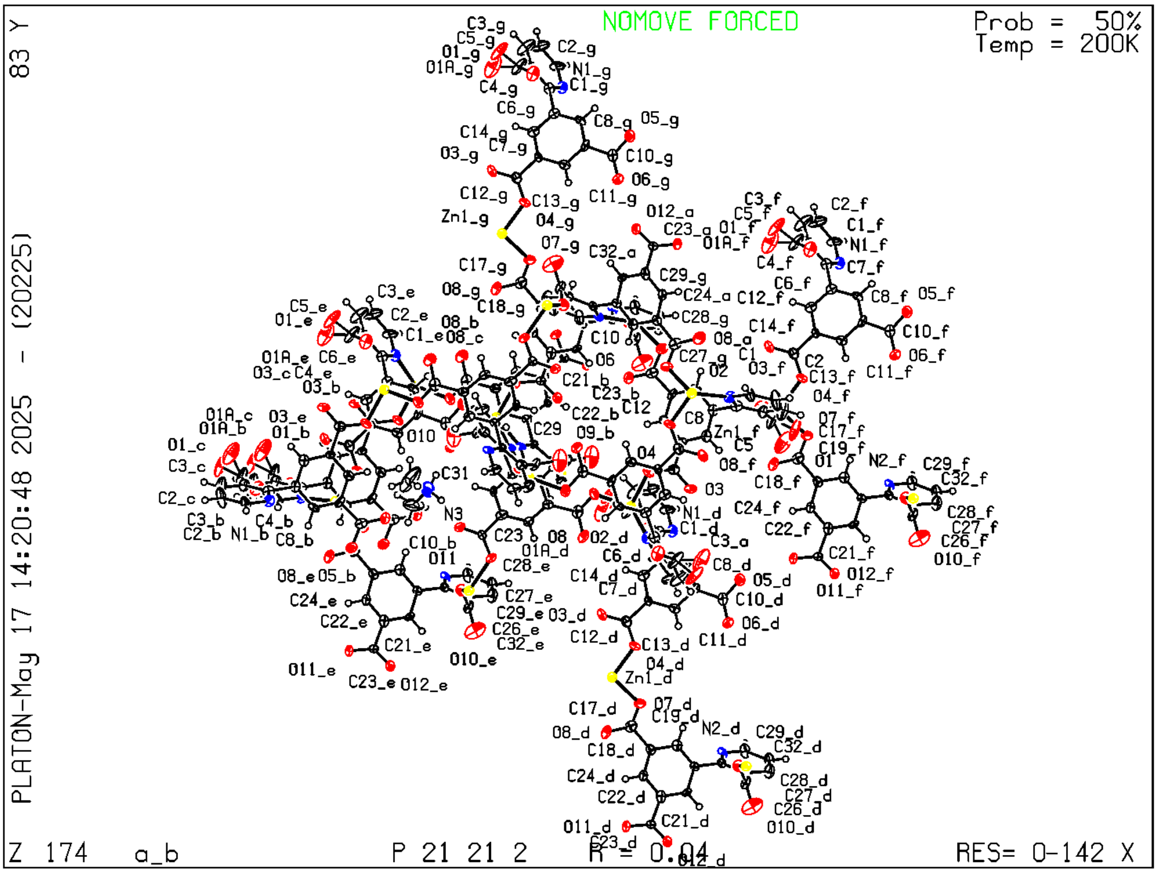

Supplement: Supplementary file 6 — Supporting Information [file ADVS-12-e09013-s002.zip › IMOF-DMF.pdf]
